# The Tbx20-TLE interaction is essential for the maintenance of the second heart field

**DOI:** 10.1242/dev.201677

**Published:** 2023-10-30

**Authors:** Whitney Edwards, Olivia K. Bussey, Frank L. Conlon

**Affiliations:** ^1^ Department of Biology and Genetics, McAllister Heart Institute, University of North Carolina at Chapel Hill, Chapel Hill, NC 27599, USA; ^2^ Integrative Program for Biological & Genome Sciences, University of North Carolina at Chapel Hill, Chapel Hill, NC 27599, USA; ^3^ Lineberger Comprehensive Cancer Center, University of North Carolina at Chapel Hill, Chapel Hill, NC 27599, USA

**Keywords:** Tbx20, Groucho, TLE, Heart development, Second heart field, Cardiac, Mouse

## Abstract

T-box transcription factor 20 (Tbx20) plays a multifaceted role in cardiac morphogenesis and controls a broad gene regulatory network. However, the mechanism by which Tbx20 activates and represses target genes in a tissue-specific and temporal manner remains unclear. Studies show that Tbx20 directly interacts with the Transducin-like Enhancer of Split (TLE) family of proteins to mediate transcriptional repression. However, a function for the Tbx20-TLE transcriptional repression complex during heart development has yet to be established. We created a mouse model with a two amino acid substitution in the Tbx20 EH1 domain, thereby disrupting the Tbx20-TLE interaction. Disruption of this interaction impaired crucial morphogenic events, including cardiac looping and chamber formation. Transcriptional profiling of Tbx20^EH1Mut^ hearts and analysis of putative direct targets revealed misexpression of the retinoic acid pathway and cardiac progenitor genes. Further, we show that altered cardiac progenitor development and function contribute to the severe cardiac defects in our model. Our studies indicate that TLE-mediated repression is a primary mechanism by which Tbx20 controls gene expression.

## Teaser

**Summary:** The Tbx20-TLE repression complex is essential for mammalian heart development and regulates cardiac progenitor cell development.

## INTRODUCTION

Embryonic heart development requires intricate regulation of transcription factor (TF) networks (TRNs) that coordinate cardiac cell specification, maturation and progression of crucial morphological events (Akerberg and Pu, 2020; Firulli and Thattaliyath, 2002; Kathiriya et al., 2015; McCulley and Black, 2012; Stennard and Harvey, 2005). The essential requirement for cardiac TRNs is emphasized by the fact that mutations in a multitude of cardiac transcription factors are causative in congenital heart disease (CHD), the most common congenital malformation (Hoffman and Kaplan, 2002; Pierpont et al., 2018; van der Linde et al., 2011). Clinical and genetic studies provide direct evidence that mutations in T-box transcription factor 20 (TBX20) are associated with a range of cardiac abnormalities (Chen et al., 2021; Kirk et al., 2007). Loss-of-function mutations in TBX20 are associated with atrial septal defects (ASDs), valve disease, cardiomyopathy and Tetralogy of Fallot (i.e. pulmonary outflow tract obstruction, ventricular septal defect and overriding aortic root) (Huang et al., 2017; Liu et al., 2008; Monroy-Muñoz et al., 2015; Qian et al., 2008; Zhao et al., 2016). In addition, gain-of-function mutations are associated with diverse clinical phenotypes, including ASDs, valve defects and patent foramen ovale (Posch et al., 2010).

Tbx20 is expressed in several cardiac cell lineages during embryogenesis, including the first heart field (FHF) and second heart field (SHF) cardiac progenitors, endocardial cells and cardiomyocytes (Carson et al., 2000; Kraus et al., 2001). Expression of Tbx20 in all major cardiac lineages explains the findings that Tbx20 is involved in numerous developmental processes during embryonic heart development. Global loss of Tbx20 in zebrafish, *Xenopus* and mice results in similar phenotypes in which cardiac looping is impaired and hearts fail to undergo chamber formation (Brown et al., 2005; Cai et al., 2005; Singh et al., 2005; Stennard et al., 2005; Szeto et al., 2002; Takeuchi et al., 2005).

In addition, cardiac lineage-specific knockouts reveal that Tbx20 is required for the proliferation and maturation of both the myocardium and endocardial-derived primordial valves (cushions) (Boogerd et al., 2016, 2018; Cai et al., 2013). Clinical observations and animal model studies highlight an essential and evolutionarily conserved function for Tbx20 in cardiac development. However, the mechanisms by which Tbx20 orchestrates these diverse developmental processes during cardiogenesis are enigmatic.

Identifying the molecular mechanisms by which Tbx20 regulates heart development is complex because Tbx20 acts as both a transcriptional activator and a repressor (Sakabe et al., 2012). Tbx20 interacts with a network of protein complexes that dictate its transcriptional activity in a temporal and context-dependent manner. *In vitro* studies show that Tbx20 interacts with a network of cardiac TFs, including Tbx5, Nkx2.5, Gata4 and Casz1, to synergistically regulate cardiac gene expression (Brown et al., 2005; Kennedy et al., 2017; Stennard et al., 2003). However, only a few interactions have been confirmed and characterized *in vivo*. Furthermore, the complete network of proteins that Tbx20 interacts with during embryonic heart development remains elusive.

Kaltenbrun et al. used an unbiased proteomics approach to identify a comprehensive Tbx20 interactome. These studies suggest that Tbx20 mediates transcriptional repression of downstream target genes via its interaction with Transducin-like Enhancer of Split (TLE) proteins, a family of transcriptional corepressors (Kaltenbrun et al., 2013). The TLE family members are the vertebrate orthologs of the *Drosophila* Groucho (Gro) protein. TLE/Gro family members perform essential functions in diverse developmental processes through their interaction with a myriad of transcription factor families (i.e. Hes, Runx, Nkx and Fox) (Agarwal et al., 2015; Chen and Courey, 2000; Cinnamon and Paroush, 2008; Fisher and Caudy, 1998; Paroush et al., 1994). TLE/Gro proteins are proposed to mediate transcriptional repression by multiple mechanisms, including the recruitment of chromatin remodeling proteins such as histone deacetylases (HDACs) to target gene loci (Chen et al., 1999; Kaltenbrun et al., 2013). Tbx20 was shown to interact directly with TLE1/3 via an evolutionarily conserved N-terminal engrailed homology (EH1) binding motif. In addition, the Tbx20-TLE interaction mediates the recruitment of chromatin remodeling proteins, including several members of the nucleosome remodeling and deacetylase complex (NuRD) (i.e. Mta1, Rbbp4, Rbbp7 and Hdac2). Further, the study by Kaltenbrun et al. demonstrated that the Tbx20-TLE complex was required for transcriptional repression during *Xenopus* embryogenesis (Kaltenbrun et al., 2013). This study provides evidence that the Tbx20-TLE interaction mediates transcriptional repression; however, the function and requirement for this interaction in cardiac development have not been elucidated.

To investigate the function of the Tbx20-TLE complex in cardiogenesis, we generated a novel mouse model in which a two amino acid substitution was introduced into the Tbx20 EH1 domain (Tbx20^EH1Mut^), thereby disrupting the Tbx20-TLE interaction. Using this model, we demonstrate that the Tbx20-TLE interaction is essential for embryonic heart morphogenesis, as mutant mice displayed impaired cardiac looping and chamber formation, resulting in embryonic lethality. Our transcriptional profiling of Tbx20^EH1Mut^ hearts and analysis of Tbx20 direct targets revealed misexpression of the retinoic acid (RA) pathway and cardiac progenitor genes, which implied that the Tbx20-TLE interaction inhibits cardiac progenitor programs in the developing heart. In addition, our studies suggest that altered cardiac progenitor specification and impaired cardiomyocyte differentiation significantly contribute to Tbx20^EH1Mut^ cardiac defects. Collectively our studies define a function for the Tbx20-TLE interaction in the developing heart and suggest that a TLE-mediated repression program is a primary mechanism by which Tbx20 regulates heart development.

## RESULTS

### The Tbx20-TLE complex is required for cardiac development

**Fig. 1.**
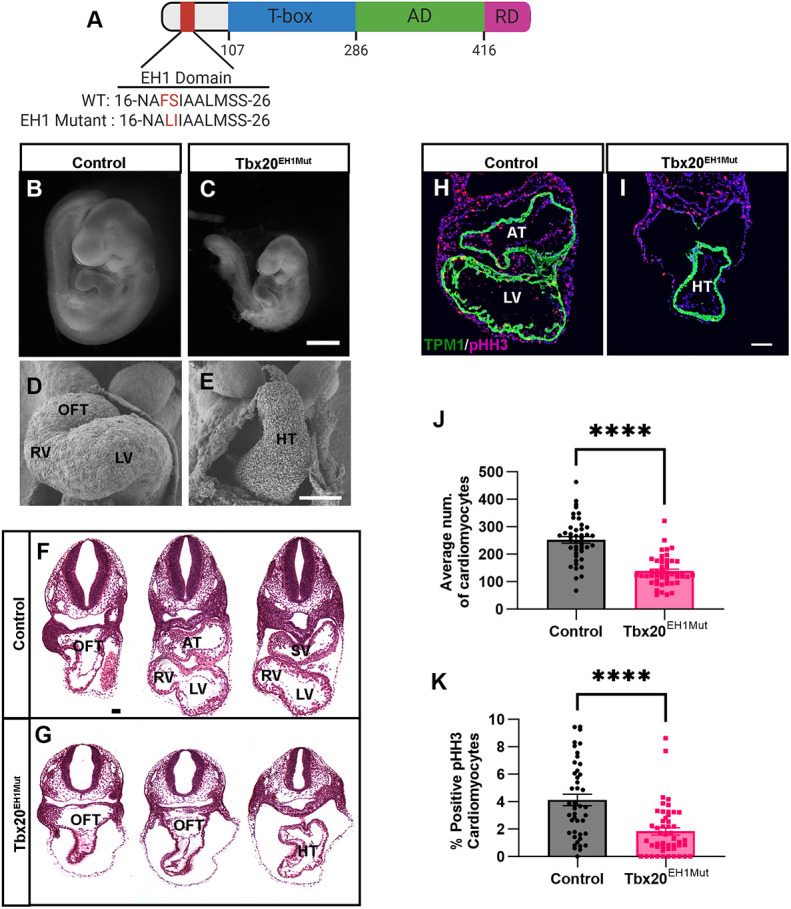
**The Tbx20-TLE complex is required for cardiac development.** (A) Schematic of Tbx20 protein, showing the wild-type and mutant engrailed homology (EH1), T-box, activation (AD) and repression (RD) domains. (B,C) Whole-mount imaging at E9.5 showing growth retardation and altered cardiac morphology in Tbx20^EH1Mut^ embryos (C) compared with control (B). *N*≥3 per genotype. (D,E) Scanning electron microscopy analysis at E9.5 reveals impaired cardiac looping and chamber formation of Tbx20^EH1Mut^ hearts. 400× magnification. *N*=2 per genotype. (F,G) Hematoxylin and Eosin-stained transverse sections representing the anterior, middle and posterior regions of E9.5 control and Tbx20^EH1Mut^ hearts. *N*=3 per genotype. (H,I) Immunohistochemical analysis of phosphohistone-H3 (pHH3)-positive cardiomyocytes (Tropomyosin; TPM1) reveals a significant decrease in the cardiomyocyte mitotic index in Tbx20^EH1Mut^ hearts. (J) Quantitation of the average number of cardiomyocytes in control and Tbx20^EH1Mut^ hearts at E9.5 (*N*=3-4 per genotype, *n*=11-16 sections analyzed per animal). (K) Quantitation of cardiomyocyte mitotic index in control and Tbx20^EH1Mut^ hearts at E9.5 (*N*=3-4 per genotype, *n*=11-16 sections analyzed per animal). Data are mean±s.e.m. ****P≤0.0001 (Welch's *t*-test). AT, atria; HT, heart tube; LV, left ventricle; OFT, outflow tract; RV, right ventricle; SV, sinus venous. Scale bars: 500 µm (B,C); 66 µm (F,G); 100 µm (D,E,H,I).

In previous studies, we demonstrated that a two amino acid substitution (phenylalanine 18 and serine 19 to leucine and isoleucine, respectively) was sufficient to specifically disrupt the Tbx20-TLE interaction and impair the recruitment of chromatin remodeling proteins (Kaltenbrun et al., 2013). To determine the *in vivo* relevance of the Tbx20-TLE interaction in the developing heart, we used CRISPR/CAS9 genome editing to introduce the same mutation in the mouse germline (Fig. 1A). Mice heterozygous for the EH1 amino acid substitutions (Tbx20^+/EH1Mut^) were viable, fertile and phenotypically indistinguishable from control littermates. In contrast, we failed to recover homozygous (Tbx20^EH1Mut^) mice postnatally (Fig. S1C). Immunohistochemical analysis showed that Tbx20 was expressed in the heart and localized to the nucleus in wild-type and Tbx20^EH1Mut^ embryos (Fig. S1A,B). In addition, mutation of the Tbx20 EH1 domain did not appear to affect binding of Tbx20 to other crucial cardiac transcription factors such as Nkx2.5 (Fig. S1D,E) (Stennard et al., 2003).

Gross morphological analysis of Tbx20^EH1Mut^ embryos at embryonic day (E)9.5 revealed that mutant embryos displayed pericardial edema and hemorrhaging (Fig. 1B,C). Ultrastructural imaging analysis (scanning electron microscopy; SEM) showed that Tbx20^EH1Mut^ hearts initiated heart tube formation but failed to undergo cardiac looping or cardiac chamber formation (Fig. 1D,E). Further, in contrast to wild-type embryos, Tbx20^EH1Mut^ hearts failed to form identifiable cardiac regions or chambers, including the outflow tract (OFT), left and right ventricles, and atria (Fig. 1F,G). Moreover, the Tbx20^EH1Mut^ cardiac phenotype was accompanied by a significant decrease in cardiomyocyte number (Tropomyosin^+^; TPM1^+^) and a concomitant decrease in the mitotic index (pHH3^+^/TPM1^+^) (Fig. 1H-K). In addition, although endocardial cells were present in the mutant heart, the endocardial cushions were not identifiable, suggesting that impaired endocardial cell development may contribute to the Tbx20^EH1Mut^ phenotype (Fig. 1G; Fig. S1B). Taken together, these data revealed that the Tbx20-TLE interaction is required for heart formation and viability.

### TBX20-TLE complex represses RA signaling genes

**Fig. 2.**
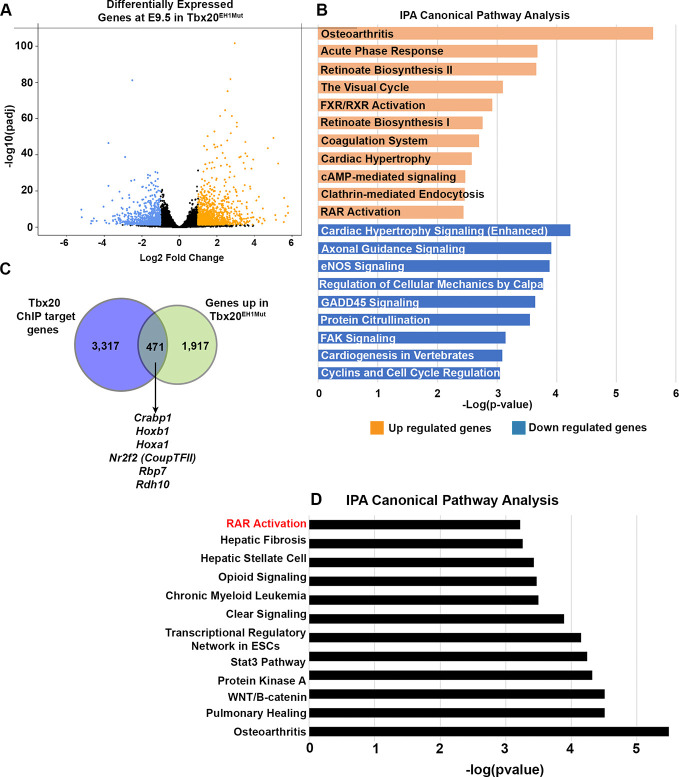
**TBX20-TLE complex represses retinoic acid signaling genes.** (A) Volcano plot of genes identified by RNA-seq to be differentially expressed between wild-type and Tbx20^EH1Mut^ hearts at E9.5 (adjusted *P*-value≤0.05 and log2 fold change≥±1). Downregulated genes are shown in blue and upregulated genes are shown in orange. (B) Ingenuity pathway analysis (IPA) of differentially expressed genes reveals misexpression of RA-associated genes in Tbx20^EH1Mut^ hearts. (C) Overlap of upregulated genes in Tbx20^EH1Mut^ hearts (adjusted *P*-value ≤0.05 and log2 fold change ≥0.585) with Tbx20 ChIP-seq dataset (Boogerd et al., 2016). (D) A subset of genes upregulated in Tbx20^EH1Mut^ hearts and identified as putative direct Tbx20 targets by ChIP-seq are associated with RA signaling as assessed by IPA.

TLE proteins function as transcriptional corepressors; therefore, we hypothesized that the Tbx20-TLE interaction represses inappropriate gene expression in the developing heart (Agarwal et al., 2015; Chen and Courey, 2000; Chen et al., 1999; Cinnamon and Paroush, 2008). To test this hypothesis, we conducted transcriptional profiling (RNA-seq) on wild-type and Tbx20^EH1Mut^ hearts at E9.5. This analysis identified 2218 differentially expressed genes (DEGs; adjusted *P*-value≤0.05 and log2 fold change≥±1), of which 1363 genes were significantly upregulated and 855 genes were significantly downregulated in Tbx20^EH1Mut^ hearts compared with controls (Fig. 2A). We performed Ingenuity Pathway Analysis (IPA) to assess the molecular pathways associated with these DEGs (Fig. 2B). Pathways associated with downregulated genes included ‘cardiac hypertrophy signaling’ and ‘factors promoting cardiogenesis in vertebrates’, all of which contain genes involved in cardiomyocyte function and growth (*Myh6*, *Nppa*, *Scn5a*, *Gja5*, *Hand1*, *Bmp2*). In addition, the ‘cyclins and cell cycle regulation’ pathway included several genes involved in cell cycle progression and proliferation (*ccnb1*, *ccnd1*, *ccnd2*). Downregulation of these pathways corroborated our findings that cardiomyocyte number and mitotic index were decreased in Tbx20^EH1Mut^ hearts.

We hypothesize that Tbx20 recruits TLE family members to target genes to mediate transcriptional repression during embryonic heart development. Therefore, we are particularly interested in investigating genes and pathways aberrantly upregulated in Tbx20^EH1Mut^ hearts. Surprisingly, analysis of upregulated genes identified several pathways associated with RA signaling, including ‘Retinoate Biosynthesis I’, ‘Retinoate Biosynthesis II’ and ‘RAR Activation’. Genes within these biological categories included the RA-synthesizing enzyme *Aldh1a2*, several RA binding proteins, *Rbp1*, *Rbp2*, *Rbp7*, and retinol dehydrogenase *Rdh12*. We confirmed that *Aldh1a2* expression is significantly increased in Tbx20^EH1Mut^ hearts compared with controls using fluorescence *in situ* hybridization (RNA-FISH) (Fig. S2A-G). These findings are particularly interesting because the RA signaling pathway is essential for cardiac morphogenesis, and perturbations in this pathway are associated with CHD, including malformations of the OFT, septal defects and cardiac looping defects (De Bono et al., 2018; Nakajima, 2019; Niederreither et al., 2001; Perl and Waxman, 2020; Rochais et al., 2009a; Ryckebusch et al., 2008; Stefanovic and Zaffran, 2017).

To determine which subset of our differentially expressed genes are putative direct targets of Tbx20, we intersected our RNA-seq data (adjusted *P*-value≤0.05 and log2 fold change≥±0.585) with published Tbx20 chromatin immunoprecipitation with high throughput sequencing (ChIP-seq) data generated from embryonic hearts (Boogerd et al., 2016). We identified 386 downregulated genes with putative Tbx20 binding sites. IPA revealed that these genes are associated with pathways involved in myocardial chamber formation and growth (‘cardiac hypertrophy signaling’), paralleling our previous results. Many of the genes are known to be specifically important for atrial/sinus venosus and atrioventricular canal development (*Tbx5*, *Gja5*, *Scn5a*, *Hcn4*, *Bmp2*). In addition, we found genes associated with cardiomyocyte energy metabolism and contractility (*Myh7*, *Myocd*, *Mybpc3*, *Atp2a2*) (Fig. S3A,B). It is hard to assess whether the Tbx20-TLE complex is involved in the direct regulation of these genes or if their altered gene expression reflects the grossly impaired chamber development and maturation of the heart tube observed in Tbx20^EH1Mut^ embryos. We next investigated genes upregulated in Tbx20^EH1Mut^ hearts, and found that 471 genes were upregulated in the mutant and putatively bound by Tbx20 (Fig. 2C). IPA analysis of these putative direct targets again revealed genes associated with RA signaling (Fig. 2D). This subset of genes included the retinol dehydrogenase *Rdh10*, RA binding proteins (*Crabp1*, *Rbp7*), RA responsive genes (*Hoxb1*, *Hoxa1*, *Nr2f2*). Based on our findings, we propose that the Tbx20-TLE complex suppresses aberrant RA signaling during embryonic heart development.

### Cardiac progenitors arrest in development in Tbx20^EH1Mut^ hearts

Misexpression of RA pathway genes in Tbx20^EH1Mut^ hearts was unexpected because these genes are primarily expressed in the SHF cardiac progenitors located outside the primary heart tube at E9.5. In combination with impaired looping and chamber formation defects in Tbx20^EH1Mut^ hearts, we suspected that this finding indicated arrested cardiomyocyte development.

**Fig. 3.**
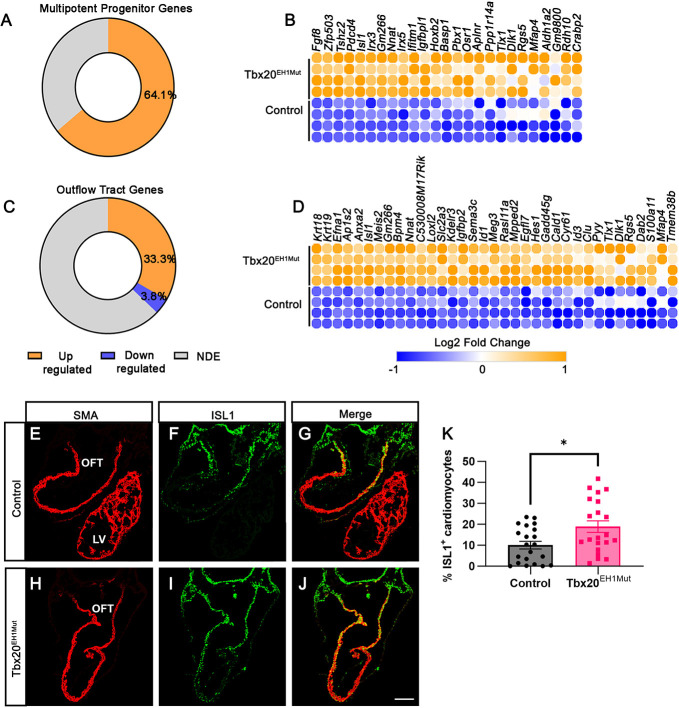
**Cardiac progenitors are arrested in development in Tbx20^EH1Mut^ hearts.** (A) Percentage of differentially expressed multipotent progenitor-associated genes in Tbx20^EH1Mut^ hearts. (B) Heatmap comparing expression of multipotent progenitor-associated genes in Tbx20^EH1Mut^ and control hearts. (C) Percentage of differentially expressed OFT-associated genes in Tbx20^EH1Mut^ hearts. (D) Heatmap comparing expression of OFT-associated genes in Tbx20^EH1Mut^ and control hearts. (E-J) Immunohistochemical analysis shows a significant increase in the percentage of Islet1 (ISL1; green)-positive cardiomyocytes (smooth muscle actin; SMA; red) in Tbx20^EH1Mut^ hearts compared with controls. (K) Quantitation of Isl1-positive cardiomyocytes in control and Tbx20^EH1Mut^ hearts at E9.5 (*N*=3 per genotype, *n*=7 sections analyzed per animal). Data are mean±s.e.m. *P≤0.05 (Welch's *t*-test). LV, left ventricle; NDE, not differentially expressed; OFT, outflow tract. Scale bar: 100 µm.

To investigate this possibility, we overlapped our gene expression dataset with a subset of genes specifically enriched in multipotent cardiac progenitors (MP), previously identified by single-cell RNA-seq (de Soysa et al., 2019). We found that ∼64% of MP-enriched genes (25/39) displayed differential expression, all of which are overexpressed in the Tbx20^EH1Mut^ heart tube (Fig. 3A). These overexpressed genes included *Isl1*, *Fgf8* and *Osr1*, well-established markers of the cardiac progenitor population (Fig. 3B) (Cai et al., 2003; Gao et al., 2019; Ilagan et al., 2006; Kang et al., 2009; Park et al., 2006; Ren et al., 2021; Sirbu et al., 2008; Snarr et al., 2007; Stefanovic and Zaffran, 2017; Xie et al., 2012; Zhang et al., 2016; Zhou et al., 2015). These data imply that the Tbx20-TLE interaction represses the expression of cardiac progenitor genes within the developing heart tube, thereby promoting the proper timing of cardiomyocyte differentiation.

### The Tbx20-TLE complex regulates SHF-derived cells

During cardiac development, SHF progenitors migrate to the anterior and posterior poles of the developing heart tube and give rise to the OFT, right ventricle and arterial poles (Rochais et al., 2009a). The RA signaling pathway regulates the development of OFT. Our finding that this pathway was upregulated in Tbx20^EH1Mut^ hearts led us to query whether the Tbx20-TLE interaction regulates OFT gene expression. Therefore, we overlapped our gene expression dataset with genes specifically enriched in the OFT during embryonic heart development. Our analyses revealed that ∼33% (35/105) of OFT-enriched genes were specifically upregulated in Tbx20^EH1Mut^ hearts (Fig. 3C). Upregulated genes included *Isl1*, *Sema3c*, *Meis2* and *Hes1*, essential mediators of OFT development (Fig. 3D) (Cai et al., 2003; Feiner et al., 2001; Rochais et al., 2009b).

Our analysis of MP- and OFT-enriched genes identified upregulation of *Isl1* in Tbx20^EH1Mut^ hearts. Isl1, a marker of both the SHF progenitors and the OFT, is a regulator of cardiac progenitor proliferation, migration and survival, and Isl1 was identified as a direct target of Tbx20 (Cai et al., 2003; Ren et al., 2021). Isl1 is highly expressed in SHF progenitors, its expression is maintained as myocardial progenitors integrate into the forming heart tube, and expression is eventually downregulated as cells begin to differentiate. To assess and validate the overexpression of Isl1, we performed an immunohistochemical analysis of control and Tbx20^EH1Mut^ hearts at E9.5. In control hearts, Isl1^+^ cardiomyocytes (Isl1^+^SMA^+^) were primarily located in the proximal OFT (Fig. 3E-G). In contrast, the number of Isl1^+^SMA^+^ cells expanded throughout the heart tube in Tbx20^EH1Mut^ hearts (Fig. 3H-J). Furthermore, we observed an almost twofold increase in the percent of Isl1^+^SMA^+^ cardiomyocytes in Tbx20^EH1Mut^ hearts compared with controls (Fig. 3K). These findings suggest that SHF-derived cells fail to undergo differentiation and remain in a cardiac progenitor-like state.

### TLE family members are expressed in the SHF during embryonic heart development

Perturbations of SHF progenitors result in impaired heart tube elongation and cardiac looping defects, similar to the effects we observed in Tbx20^EH1Mut^ mice (Kang et al., 2009; Prall et al., 2007; Vong et al., 2006). Previous studies have demonstrated that *Tbx20* is expressed in the SHF progenitors during embryonic heart development (Kraus et al., 2001; Takeuchi et al., 2005). Therefore, we hypothesized that the Tbx20-TLE interaction regulates the SHF progenitor population. To address this hypothesis, we first examined the expression of TLE family members in the SHF during embryonic heart development.

**Fig. 4.**
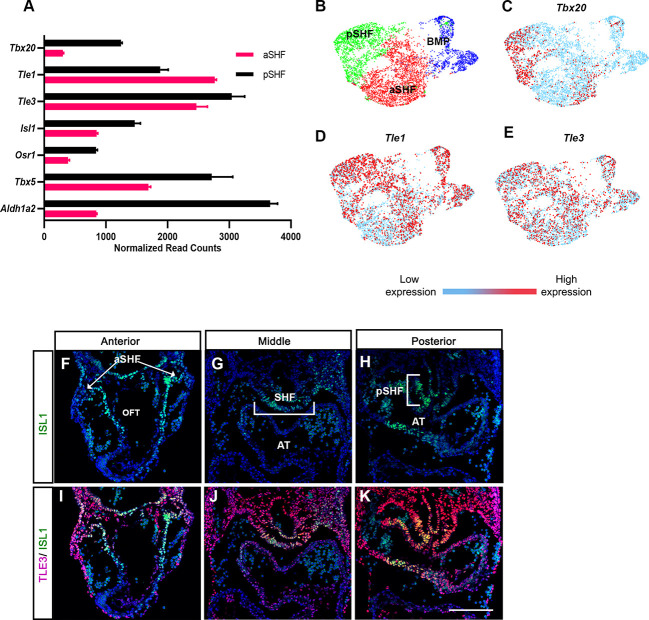
**TLE family members are expressed in the second heart field during embryonic heart development.** (A) Relative transcript abundance of *Tbx20*, *Tle1*, *Tle3* and SHF-associated genes from RNA-seq analysis of anterior and posterior SHF progenitor populations (Stefanovic et al., 2020). (B) Uniform manifold approximation and project (UMAP) plot of cardiac progenitor subpopulations generated from single-cell RNA-seq analysis of embryonic hearts (de Soysa et al., 2019). (C-E) UMAP plot of *Tbx20*, *Tle1* and *Tle3* in cardiac progenitor subpopulations. (F-K) Immunohistochemical co-expression analysis of Isl1 (green) and Tle3 (magenta) shows that Tle3 is robustly expressed in the SHF of control embryos at E9.5. aSHF, anterior second heart field; AT, atria; BMP, branchiomeric muscle progenitors; OFT, outflow tract; pSHF, posterior second heart field. Scale bar: 100 µm.

The SHF can be subdivided into two distinct regions. Progenitors derived from the anterior SHF (aSHF) give rise to the right ventricle and portions of the OFT. The posterior SHF (pSHF) progenitors give rise primarily to the atria and a subset of cardiac vessels (Bertrand et al., 2011; Domínguez et al., 2012; van den Berg et al., 2009). Analysis of published transcriptomic profiling of the aSHF and pSHF at E9.5 revealed that *Tle1* and *Tle3* are highly expressed in both SHF domains (Fig. 4A) (Stefanovic et al., 2020). *Tbx20* was also detected in both SHF populations; however, its expression pattern more closely paralleled well-established pSHF makers (*Aldh1a2*, *Tbx5* and *Osr1*) as we observed higher expression in the pSHF compared with the aSHF (Fig. 4A). We obtained similar results from a recent single-cell RNA-seq analysis of the *Isl1*^+^ SHF progenitor population at the early stages of embryonic heart development (Fig. 4B-E) (de Soysa et al., 2019). In addition, immunohistochemical analysis of wild-type embryos at E9.5 validated the expression of TLE family members in the SHF. Our analysis demonstrated that Tle3 was highly expressed in both domains of the SHF and colocalized with the pan-SHF marker Isl1 in the majority of SHF cells (Fig. 4F-K). Together, these data demonstrate that *Tbx20*, *Tle1* and *Tle3* are expressed in SHF progenitors and therefore support a role for the Tbx20-TLE complex in this cardiac progenitor population.

### Tbx20-TLE complex regulates cardiac progenitor specification

**Fig. 5.**
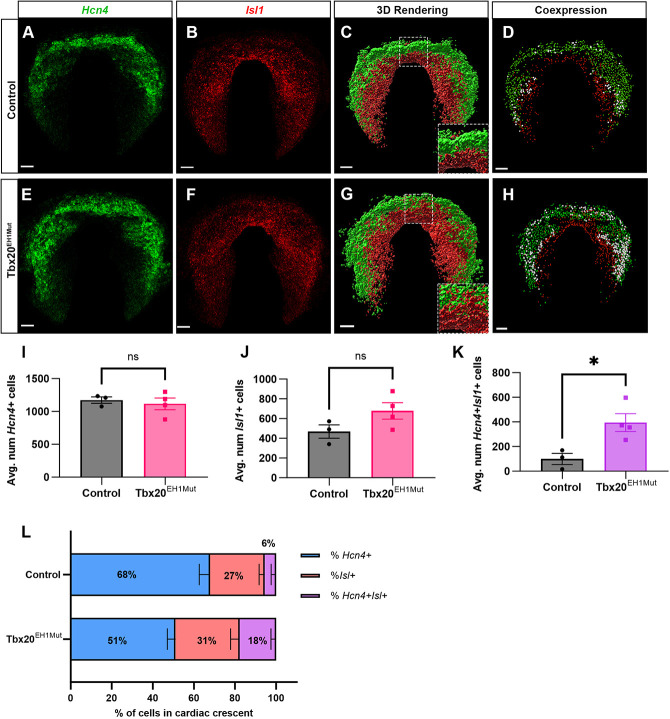
**Tbx20-TLE complex regulates cardiac progenitor specification.** (A,B) WMFISH analysis of *Hcn4* (FHF; green) and *Isl1* (SHF; red) in the cardiac crescent (CC) of control embryos at E7.75. (C) 3D reconstruction of *Hcn4* and *Isl1* surfaces in control embryos at E7.75. (D) 3D reconstruction of *Hcn4*^+^ (green dots) and *Isl1*^+^ (red dots) cells in control embryos at E7.75. Cells co-expressing *Hcn4* and *Isl1* are represented by white dots. (E,F) WMFISH analysis of *Hcn4* and *Isl1* in the CC of Tbx20^EH1Mut^ embryos at E7.75. (G) 3D reconstruction of *Hcn4* and *Isl1* surfaces shows altered spatial organization of FHF and SHF in Tbx20^EH1Mut^ embryos at E7.75. (H) 3D reconstruction of *Hcn4*^+^ and *Isl1*^+^ cells in Tbx20^EH1Mut^ embryos at E7.75. The number of cells co-expressing *Hcn4* and *Isl1* (white dots) are increased in Tbx20^EH1Mut^ embryos compared with controls. (I) Quantitation of *Hcn4*^+^ cells in the CC of control and Tbx20^EH1Mut^ embryos at E7.75. *N*=3-4 per genotype. (J) Quantitation of *Isl1*^+^ cells in the CC of control and Tbx20^EH1Mut^ embryos at E7.75. *N*=3-4 per genotype. (K) Quantitation of cells co-expressing *Hcn4* and *Isl1* in the CC of control and Tbx20^EH1Mut^ embryos at E7.75. *N*=3-4 per genotype. (L) Relative proportions (expressed as a percentage) of *Hcn4*^+^ (blue), *Isl1*^+^ (red) and *Hcn4*^+^*Isl1*^+^ (purple) in the CC of control and Tbx20^EH1Mut^ embryos at E7.75. Data are mean±s.e.m. *P≤0.05 (Welch's *t*-test). Scale bars: 50 µm.

In combination, our findings of impaired elongation and looping, upregulation of cardiac progenitor and OFT-associated genes in the heart tube, and detection of TLE family members in the SHF suggested that the SHF progenitor population may be affected in Tbx20^EH1Mut^ embryos. The primary heart tube is derived from FHF cells, an additional pool of cardiac progenitors. Intriguingly, we found hallmark genes of the FHF, including *Hcn4*, *Gata4* and *Hand1*, were downregulated in Tbx20^EH1Mut^ hearts (Fig. S4A-C) (Barnes et al., 2010; Liang et al., 2013; Zhang et al., 2021; Zhao et al., 2008). These observations suggest that alterations in the FHF and SHF progenitor populations may contribute to the impaired heart development in the mutants. To determine whether the FHF and SHF progenitors are altered in the Tbx20^EH1Mut^ embryos, we performed whole-mount fluorescent *in situ* hybridization (WMFISH). We conducted a quantitative spatial analysis of the cardiac crescent (CC) at E7.75. In control embryos, FHF progenitors, identified by expression of *Hcn4*, reside within the CC, whereas *Isl1*^+^ SHF progenitors lie medial to the CC (Fig. 5A-C). The FHF and SHF populations are spatially segregated, and we detected relatively few *Hcn4* and *Isl1* double-positive cells (*Hcn4*^+^*Isl1*^+^) in control embryos (Fig. 5D,K). Tbx20^EH1Mut^ embryos displayed no apparent differences in the localization or number of FHF progenitors (Fig. 5E,I). Conversely, although the number of *Isl1*^+^ cells was not significantly changed, the spatial positioning of the SHF cells appeared to be disrupted, as we observed a ventrolateral displacement of *Isl1*^+^ cells (Fig. 5F,G,J). This change in localization was most apparent in the anterior position of the CC (Fig. 5C,G, insets). Unexpectedly, we also observed a drastic increase in the number (∼fourfold) and proportion of *Hcn4*^+^*Isl1*^+^ in the mutant embryos (Fig. 5H,K,L). These findings indicate that a subset of cardiac progenitors in Tbx20^EH1Mut^ embryos were misspecified and fail to adopt an FHF or SHF identity.

### Tbx20-TLE is essential for the maintenance of the SHF

**Fig. 6.**
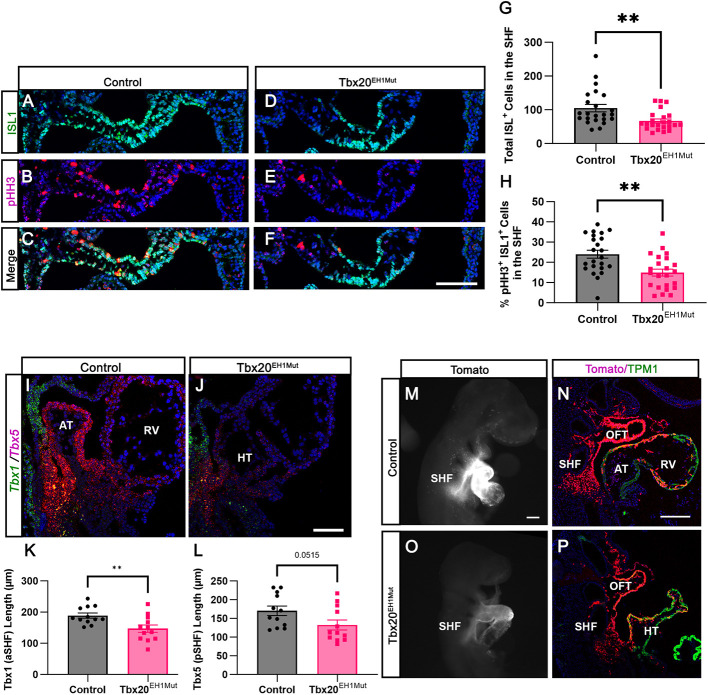
**Tbx20-TLE is essential for the maintenance of the SHF.** (A-F) Immunohistochemical analysis of phosphohistone-H3 (pHH3; red) and Isl1 (green) expression in the SHF shows a significant decrease in the mitotic index of Isl1^+^ cells in the SHF of Tbx20^EH1Mut^ embryos at E9.5. (G) Quantitation of the total number of Isl1^+^ cells in the SHF of control and Tbx20^EH1Mut^ embryos (*N*=4 per genotype, *n*=5-7 sections per animal). (H) Quantitation of mitotic index of Isl1^+^ cells in the SHF of control and Tbx20^EH1Mut^ embryos (*N*=4 per genotype, *n*=5-7 sections per animal). (I,J) RNA-FISH analysis of *Tbx1* (aSHF) and *Tbx5* (pSHF) shows a decrease in the anterior and posterior SHF domains in Tbx20^EH1Mut^ embryos compared with controls at E9.5. (K) Quantitation of *Tbx1* (aSHF) domain in control and Tbx20^EH1Mut^ embryos (*n*=4 sections per animal, *N*=3 per genotype). (L) Quantitation of *Tbx5* (pSHF) domain in control and Tbx20^EH1Mut^ embryos (*n*=4 sections per animal, *N*=4 per genotype). (M,O) Whole-mount fluorescent imaging of *Mef2c*-AHF genetic lineage tracing (Tomato fluorescence is pseudo-colored white in M,O) shows a reduced SHF progenitor population in Tbx20^EH1Mut^ embryos compared with controls. (N,P) Representative sagittal sections of *Mef2c*-AHF genetic lineage tracing (red) in control and Tbx20^EH1Mut^ embryos. Sections are co-stained with TPM1 (green). Data are mean±s.e.m. **P≤0.01 (Welch's *t*-test). aSHF, anterior second heart field; AT; atria; HT, heart tube; OFT, outflow tract; pSHF, posterior second heart field; RV, right ventricle; SHF, second heart field. Scale bars: 100 µm (A-F,I,J,M,O); 200 µm (N,P).

Given that cardiac progenitors are perturbed in Tbx20^EH1Mut^ embryos at E7.75, we also wanted to assess whether progenitor dysfunctions persisted at later stages of development. By E9.5, the FHF progenitor pool had differentiated and formed the heart tube (Buckingham et al., 2005; Kelly and Buckingham, 2002; Kelly et al., 2014); therefore, we focused on the reservoir of remaining SHF progenitors. To determine whether the SHF was altered in E9.5 Tbx20^EH1Mut^ embryos, we first quantified the expression of Isl1, a marker for both the aSHF and pSHF. We found a significant reduction in the total number of Isl1^+^ cells in the SHF in Tbx20^EH1Mut^ embryos compared with wild-type controls (Fig. 6A,D,G). In addition, we determined that the mitotic index (pHH3^+^Isl1^+^) of Isl1^+^ SHF progenitors was reduced in Tbx20^EH1Mut^ embryos (Fig. 6B,C,E,F,H). These findings suggest that Tbx20^EH1Mut^ embryos have a reduction in SHF progenitors by E9.5, a time when SHF progenitors substantially contribute to the addition and elongation of the developing heart tube.

The anterior and posterior SHF progenitors have unique expression profiles, occupy distinct spatial compartments within the SHF and are controlled by different transcriptional programs (Bertrand et al., 2011; Domínguez et al., 2012; Stefanovic et al., 2020; van den Berg et al., 2009). Although our data suggest that *Tbx20* and TLE family members are expressed in both SHF populations, *Tbx20* appears to have higher expression in the pSHF (Fig. 4A-E). Therefore, we next sought to determine whether the SHF subpopulations are differentially affected by the disruption of the Tbx20-TLE interaction. Using RNA-FISH we quantified the relative length of the *Tbx1* and *Tbx5* expression domains, which have been shown to demarcate the boundaries between the aSHF and pSHF, respectively (De Bono et al., 2018). In both controls and Tbx20^EH1Mut^ embryos, we observed distinct boundaries between the aSHF (*Tbx1*) and the pSHF (*Tbx5*), although overall expression of these both genes appeared to be decreased in the mutants (Fig. 6I,J). In addition, the relative length of both the *Tbx1* and *Tbx5* domains were significantly decreased in Tbx20^EH1Mut^ embryos compared with controls (Fig. 6K,L). These findings suggest that loss of the Tbx20-TLE complex results in perturbation of both aSHF and pSHF progenitor populations.

We performed lineage tracing to examine the fate of SHF progenitors and SHF-derived cells in Tbx20^EH1Mut^ embryos. For these studies, the Tbx20^EH1Mut^ mouse line was crossed to *Mef2c-AHF-Cre*; Rosa^td^ mice, a transgenic mouse line that drives Tomato expression in a subdomain of SHF progenitors and lineages derived from this population (Verzi et al., 2005). In control embryos, tomato-positive (Td^+^) cells were primarily detected in the SHF, OFT and right ventricle (RV) (Fig. 6M,N). In Tbx20^EH1Mut^ mice, Td^+^ cells were detected mainly in the heart tube and what we presumed to be the OFT (Fig. 6O,P). These data demonstrated that at least a subset of SHF progenitors migrate and integrate into the heart tube in Tbx20^EH1Mut^ embryos. Of note, although in control embryos SHF-derived cells give rise to distinct regions of the heart, in the mutant embryo most of the cells within the heart tube are Td^+^. This suggests that the heart tube in Tbx20^EH1Mut^ embryos comprises a more significant proportion of SHF-derived cells, supporting our findings that OFT and SHF-progenitor genes are upregulated in mutant hearts (Fig. 4). Consistent with our analysis of Isl1^+^ progenitors in the SHF, we also observed a striking reduction in the SHF progenitor Td^+^ population in Tbx20^EH1Mut^ mice compared with control embryos (Fig. 6O,P). Taken together, these data suggest that aberrant specification and perturbed SHF dynamics underlie the loss of the SHF population at E9.5. Further, perturbations in this crucial progenitor population during development likely contribute to the cardiac phenotype observed in Tbx20^EH1Mut^ embryos. Our study highlights an unexplored role for the Tbx20-TLE complex in cardiac progenitor development and function.

## DISCUSSION

Tbx20 is essential for heart development, and its disease relevance is well established (Brown et al., 2005; Cai et al., 2005; Chen et al., 2021; Kirk et al., 2007; Singh et al., 2005; Stennard et al., 2005; Takeuchi et al., 2005). However, it was not known how Tbx20 mediates transcriptional regulation of an extensive network of cardiac genes. Here, we demonstrate that Tbx20 interaction with the Gro/TLE family of transcriptional corepressors is required for embryonic heart development. Disruption of the Tbx20-TLE interaction resulted in severe cardiac defects and embryonic lethality. Our findings suggest that the Tbx20-TLE complex represses progenitor gene expression in the developing heart. Further, we demonstrate that the Tbx20-TLE complex is essential for maintaining the SHF population. Our studies show that TLE proteins are an integral component of the Tbx20 interaction network, and our findings further elucidate the regulatory mechanisms of Tbx20 in the developing heart.

### Tbx20-TLE-mediated transcriptional repression

Tbx20 functions as both a transcriptional repressor and an activator, and studies indicate that its interactions with various protein networks dictate the transcriptional activities of Tbx20. Tbx20 directly interacts with crucial cardiac transcription factors, including Isl1, Gata4, Nkx2.5 and Casz1 (Brown et al., 2005; Kennedy et al., 2017; Stennard et al., 2003). However, few studies have directly tested the activities of these Tbx20 interactions *in vivo*.

More recently, Tbx20 has been shown to interact with TLE proteins, a family of transcriptional corepressors (Kaltenbrun et al., 2013). Further, the Tbx20-TLE complex is proposed to mediate transcriptional repression via the recruitment of the NuRD components (Kaltenbrun et al., 2013). Our current study aimed to investigate the requirement for the Tbx20-TLE interaction during mammalian heart development. We generated a mouse model with a two amino acid substitution in the Tbx20 EH1 domain, disrupting the Tbx20-TLE complex (Tbx20^EH1Mut^). We found that Tbx20^EH1Mut^ mice had severely impaired heart formation and were arrested in development at E9.5. These findings demonstrated that the Tbx20-TLE repression complex is crucial for embryonic heart development.

The cardiac defects in the Tbx20^EH1Mut^ mouse phenotypically parallel the Tbx20 null and global loss-of-function mutants (Cai et al., 2005; Singh et al., 2005; Stennard et al., 2005; Takeuchi et al., 2005). These models and ours display severely hypoplastic hearts that fail to undergo looping/chamber formation, resulting in embryonic lethality. Although the specific genes reported to be misregulated differ across the various Tbx20 null mutant mouse models, it is apparent that the genetic programs controlling chamber specification and maturation are severely dysregulated. These models established an essential role for Tbx20 in cardiac chamber development. However, these studies were unable to decipher whether Tbx20 directly activates genes involved in chamber differentiation or functions to repress genetic programs that impede chamber formation. Our model suggests the latter mechanism, as we also observed that genes involved in atrial and ventricular chamber formation (*Nppa*, *Hcn4*, *Tbx5*, *Hand1*) are downregulated in the Tbx20^EH1Mut^ heart. Therefore, Tbx20-TLE-mediated repression may serve as a primary mechanism by which Tbx20 regulates cardiac gene expression.

Our study supports a crucial role for Tbx20 repression complexes in heart development. However, relatively little is known about the cardiac gene network that is regulated by Tbx20-mediated repression. To investigate the molecular underpinnings of impaired cardiac development in Tbx20^EH1Mut^ and to identify the subset of cardiac genes repressed by Tbx20, we performed transcriptomics analysis. We intersected our dataset with ChIP-seq data to identify putative direct Tbx20 targets (Boogerd et al., 2016). We found that several genes associated with the RA pathway (*Aldh1a2*, *Hoxa1*, *Hoxb1*, *Cyp26a1*) were upregulated in Tbx20^EH1Mut^ hearts. Although regulation of the RA genes by Tbx20 was not demonstrated previously, Tbx5 and Tbx1, two additional T-box family members, are known regulators of the RA signaling genes (De Bono et al., 2018; Guris et al., 2006; Takahashi and Yamanaka, 2006). Cardiac defects observed with loss of Tbx1 are associated with aberrant activation of the RA signaling pathway (Aggarwal and Morrow, 2008; Guris et al., 2006; Parisot et al., 2011; Roberts et al., 2006; Ryckebusch et al., 2010; Yutzey, 2010). In addition, Rankin et al. recently demonstrated that Tbx5 directly activates the expression of *Aldh1a2* in cardiac progenitors (Rankin et al., 2021). Together with our findings, these studies suggest that T-box transcription factors are crucial regulators of the RA gene network. The RA signaling pathway has multifaceted functions in embryonic heart development and CHD. Studies across multiple species have demonstrated that proper RA titration is essential for cardiac morphogenesis, OFT development, and cardiomyocyte specification (De Bono et al., 2018; Nakajima, 2019; Niederreither et al., 2001; Rochais et al., 2009a; Ryckebusch et al., 2008; Stefanovic and Zaffran, 2017). In addition, alterations in the RA pathway are associated with cardiac looping defects (Bouman et al., 1995; Collop et al., 2006; Nakajima, 2019; Niederreither et al., 2001; Perl and Waxman, 2019, 2020; Smith et al., 1997). Therefore, our findings suggest that altered RA signaling contributes to the abnormal cardiac phenotype in Tbx20^EH1Mut^ mice.

Our transcriptomic analysis also revealed that a large group of cardiac progenitor and OFT genes were overexpressed in Tbx20^EH1Mut^ hearts. Included in this group was *Isl1*, a well-established direct transcriptional target of Tbx20 (Boogerd et al., 2018; Cai et al., 2005). In addition, we showed that the number of Isl1^+^ cardiomyocytes was significantly increased in Tbx20^EH1Mut^ hearts. These findings are corroborated by previous studies that show that loss of Tbx20 results in the upregulation of Isl1 in the myocardium (Boogerd et al., 2018; Cai et al., 2005). Furthermore, studies show that conditional loss of Tbx20 in embryonic cardiomyocytes results in upregulation of *Fgf10* and *Hopx*, progenitor genes identified as putative direct Tbx20 targets, both of which were upregulated in the Tbx20^EH1Mut^ hearts (Table S1) (Boogerd et al., 2018). During the early stages of heart development, cardiac progenitors enter the heart tube, differentiate and coincidently downregulate the progenitor transcriptional program (Dyer and Kirby, 2009; Rochais et al., 2009a; Zaffran and Kelly, 2012). Therefore, these findings suggest that the Tbx20-TLE complex mediates the repression of the cardiac progenitor gene program in the developing heart tube and indicates that impaired differentiation contributes to developmental defects in Tbx20^EH1Mut^ mice.

Paradoxically, in Tbx20^EH1Mut^ hearts, we observe an increase in cells that appear to be stuck in a progenitor-like state, coincident with a significant decrease in cardiomyocyte proliferation. We propose that this is a consequence of altered cell cycle regulation in mutant hearts. Dynamics of cardiomyocyte proliferation are rapidly changing during the early stages of embryonic heart development (Günthel et al., 2018). Early cardiac progenitors display robust proliferation, but cardiomyocyte proliferation ceases as cells begin to differentiate during primary heart tube formation. Proliferation is then reinitiated after looping and during the beginning stages of chamber formation (Günthel et al., 2018; van den Berg et al., 2009). Because we observe impaired looping/chamber formation, we propose that the decrease in proliferation observed in Tbx20^EH1Mut^ may reflect an inability to reinitiate cardiomyocyte proliferation.

### The Tbx20-TLE complex regulates the cardiac progenitors

During early embryonic development, the FHF and SHF cardiac progenitor populations give rise to differentiated cardiac cell types. Although these two populations develop simultaneously, they are molecularly, anatomically and functionally distinct (Buckingham et al., 2005; Kelly and Buckingham, 2002; Kelly et al., 2014). FHF progenitors are the first to differentiate and give rise to the primitive heart tube, and specifically contribute to the left ventricle and atria. SHF progenitors display delayed differentiation and migrate to the anterior and posterior poles of the heart after primary heart tube formation, at which time they immediately differentiate and drive tube elongation. These cells contribute primarily to the OFT, RV and atria (Dominguez et al., 2023; Kelly et al., 2014; Rochais et al., 2009a; Zaffran and Kelly, 2012).

Although the primitive heart tube forms in our model, altered expression of FHF and SHF genes suggests cardiac progenitors may be affected. In addition, impaired looping, tube elongation and OFT development specifically implicates altered SHF progenitor function (Kang et al., 2009; Prall et al., 2007; Vong et al., 2006). Interestingly, different research groups who used distinct Tbx20 null mouse models arrived at opposite conclusions regarding Tbx20 regulation of SHF progenitors (Cai et al., 2005; Singh et al., 2005; Stennard et al., 2005; Takeuchi et al., 2005). Although definitive experiments directly investigating the molecular function of Tbx20 in the SHF have not been conducted, several studies support a role for Tbx20 in SHF progenitor function and maintenance. Expression analysis shows that *Tbx20* is abundantly expressed in early cardiac progenitors located in the CC at E7.5 (Kraus et al., 2001; Takeuchi et al., 2005). In addition, *in vitro* studies demonstrate that Tbx20 functions in concert with other cardiac progenitor transcription factors to regulate the expression of SHF-associated genes (Takeuchi et al., 2005). Further, recent zebrafish studies show that loss of Tbx20 early in cardiogenesis results in impaired cardiac progenitor development (Lu et al., 2017). In agreement with these studies, our analysis of single-cell RNA-seq data showed that *Tbx20* is expressed in the SHF populations during cardiogenesis.

**Fig. 7.**
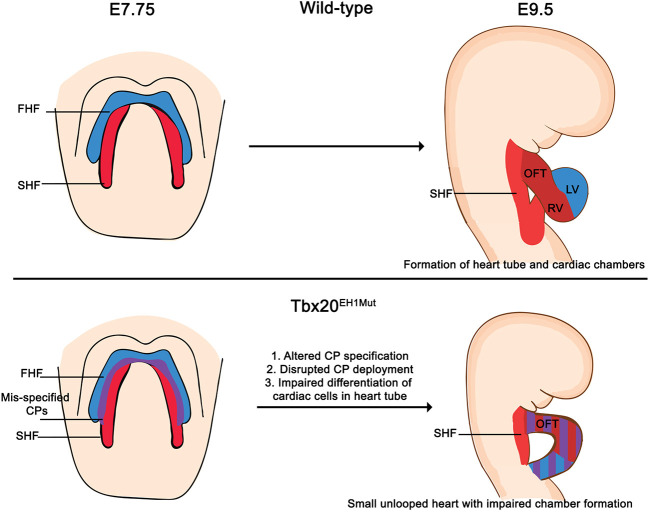
**Model describing altered cardiac development in Tbx20^EH1Mut^ mice.** Our study suggests that altered cardiac progenitor specification, disrupted progenitor deployment and impaired cardiac cell differentiation underly the impaired heart looping and chamber defects observed in Tbx20^EH1Mut^ mice. These studies show that the Tbx20-TLE transcriptional repression complex is a crucial regulator of cardiogenesis.

Our analysis of FHF and SHF progenitors in Tbx20^EH1Mut^ also supports a role for Tbx20 in regulating cardiac progenitor dynamics. At E7.75, the FHF and SHF progenitors are clearly defined in control animals. In contrast, we show cells that express both FHF and SHF makers (*Hcn4*^+^*Isl1*^+^) are substantially increased in Tbx20^EH1Mut^ embryos, indicating that the Tbx20-TLE repression complex regulates transcriptional programs in FHF and SHF progenitors. We hypothesize that impaired heart tube formation in mutants is due in part to compromised progenitor specification, which results in uncoordinated differentiation and deployment. For example, disrupted cardiac specification could account for the decrease in FHF/left ventricle-specific gene expression in Tbx20^EH1Mut^ hearts. We also propose that the reduction of the SHF pool, paralleled with the increase in the proportion of SHF-derived cells in the heart tube at E9.5, suggests premature deployment of SHF progenitors. Taken together, our study suggests that impaired heart development in Tbx20^EH1Mut^ embryos results from misspecification of cardiac progenitors and perturbed progenitor function, including premature deployment of the SHF cells. Finally, although SHF cells successfully incorporate into the heart tube, they arrest in development and are unable to differentiate further (Fig. 7). Although future studies will be necessary to elucidate underlying mechanisms, our studies are the first to show a crucial role for the TBX20-TLE repressive complex in cardiac progenitor development and function.

### Implications for Tbx20-TLE complex in CHDs

Elucidating transcriptional mechanisms is essential for understanding how Tbx20 regulates multiple cardiogenic processes. Our findings provide a new understanding of Tbx20-mediated transcriptional repression and indicate a crucial function for the Tbx20-TLE complex during heart development. Our studies also have implications for CHD and regenerative strategies. Mutations in components of the TLE repression complex or the transcriptional targets of the TBX20-TLE complex may be causative in various CHD. Our findings also suggest that cardiac phenotypes due to mutations in TBX20 or downstream target genes are not only due to loss of gene expression but also misexpression of genes in SHF-derived cells. Finally, we note that TBX20 is not only involved in CHD but is also associated with cardiomyopathy in patients (Kennedy et al., 2017; Kirk et al., 2007; Shen et al., 2011; Zhao et al., 2016). In mice, ablation of Tbx20 in adult cardiomyocytes leads to severe cardiomyopathy and premature death. Determining how abnormalities of the Tbx20-TLE complex contribute to adult-onset heart disease and identifying mutations in the TLE/Gro complex associated with cardiovascular disease will be crucial for assessing the function of the Tbx20-TLE interactions in additional disease states.

## MATERIALS AND METHODS

### Mice

Tbx20^EH1mut^ mice were generated by the UNC Animals Models Core Facility. Wild-type C57/Bl6, Gt(ROSA)26Sortm14(CAG-tdTomato)Hze (stock 007194), Mef2c-AHF-cre (Verzi et al., 2005) mice have all been previously described and were obtained from The Jackson Laboratory. Male and female mice were used in experiments. Research and animal use was approved by the Institutional Animal Care and Use Committee at the University of North Carolina and conformed to the Guide for the Care and Use of Laboratory Animals.

### Generation of Tbx20^EH1mut^ mouse line

Cas9 guide RNAs (gRNAs) targeting the *Tbx20* F18 and S19 codons were identified using Benchling software. Two gRNAs were selected for activity testing. gRNAs were cloned into a T7 promoter vector (UNC Animal Models Core) followed by *in vitro* transcription and silica spin column purification. Functional testing was performed by *in vitro* Cas9 cleavage assay. Each gRNA was incubated with recombinant Cas9 protein (UNC Animal Models Core) and PCR-amplified target region DNA, followed by gel electrophoresis to detect cleavage of the target site. Two guide RNAs were selected for genome editing in embryos: Tbx20-sg97B (protospacer sequence 5′-GGACATAAGCGCGGCGA-3′) and Tbx20-sg83B (protospacer sequence 5′-AAGCGCGGCGATGGAGA-3′). A donor oligonucleotide was designed to facilitate homologous recombination to introduce the F18L (TTC to TTa) and S19I (TCC to atC) mutations. The donor sequence was 5′-ATGGAGTTCACGGCGTCGCCCAAGCCCCAGCTCTCCTCTCGAGCCAATGCCTTaatCATCGCCGCGCTTATGTCCAGCGGCGGCCCCAAGGAGAAGGAGGCAGCAGAG -3′.

Embryos were produced by *in vitro* fertilization of C57BL/6J oocytes with sperm from a *Tbx20*^*Avi/+*^ male. Embryos were divided into two groups and each group was microinjected with recombinant Cas9 protein, donor oligo and one of the two guide RNAs. Microinjected embryos were implanted in pseudopregnant recipient females and resulting pups were screened by PCR and sequencing for the presence of the F18L-S19I allele. The first microinjection session was performed with 400 nM Cas9, 50 ng/μl gRNA and 50 ng/μl donor oligonucleotide. The session yielded three pups from 444 injected embryos, with none of the pups showing mutations at the target site. A second microinjection session was performed with 200 nM Cas9, 25 ng/μl gRNA and 50 ng/μl donor oligonucleotide. The Tbx20-sg97B gRNA mix yielded two pups from 188 injected embryos, with one animal showing insertion/deletion (indel) mutations at the target site. The Tbx20-sg83B yielded 11 pups from 291 injected embryos. One male (#7) was found to have the desired F18L-S19I amino acid changes. The animal did not have the Tbx20-avitag, indicating that the F18L-S19I mutation was inserted in an otherwise wild-type Tbx20 allele. The positive founder was transferred to the client for breeding. Two additional animals with indel mutations were also identified in this group. A third injection session was performed with the same reagent concentrations as the second, yielding three pups from the Tbx20-sg97B gRNA and nine pups from the Tbx20-sg83B gRNA. One male (#21) from the sg83B injection had a small amount of the F18L-S19I mutation in addition to an indel, and was positive for the Tbx20-avitag. The founder was mated to C57BL/6J females for transmission of the mutant alleles. Approximately 50% of the pups from this founder had a 16 bp deletion mutation at the F18-S19 target area *in cis* with the avitag insertion. The other pups were wild type at the F18-S19 target region, and no transmission of the F18L-S19I mutation was detected. The resulting pups were screened by PCR and sequenced for the presence of the mutation allele. Male founders with the correct mutation were mated to wild-type C57BL/6J females for germline transmission of the mutated allele. Lines were back-crossed for at least three generations.

### Scanning electron microscopy

SEM was performed as previously described (Dorr et al., 2015). Briefly, embryos were fixed in paraformaldehyde/2.5% glutaraldehyde in 1× PBS, washed in 1× PBS, dehydrated and subjected to critical point drying. Embryos were mounted ventral side up and ion sputtered with gold-palladium to 10 µm thickness. Embryos were scanned with a Zeiss Supra 25 FESEM microscope. SEM photomicrographs were taken in standard orientations and magnifications.

### Histology and immunohistochemistry

For histology and immunohistochemistry, embryos were fixed in 4% paraformaldehyde/PBST (PBS + 0.1% Tween) overnight at 4°C. Embryos were then processed for either paraffin embedding or frozen in OCT. For histology, paraffin sections were dewaxed and stained with Hematoxylin and Eosin, according to standard protocols (Cardiff et al., 2014). Histology sections were imaged on an Olympus BX61 fluorescence microscope. For immunohistochemistry, cryosections were thawed, washed in 1× PBS and subjected to antigen retrieval as previously described (Dorr et al., 2015). The following primary antibodies were used: mouse anti-tropomyosin [Developmental Studies Hybridoma Bank (DSHB) clone CH1, 1:50]; rabbit anti-phospho-histone H3 (Millipore, 06-570, 1:200); mouse anti-Islet1 (DSHB, clone 39.4D5, 1:75); rabbit anti-Tle3 (Abcam, ab94972, 1:500); rabbit anti-smooth muscle actin (Abcam, ab5694, 1:500); rabbit anti-Tbx20 (Genscript, GS5922, 1:250). Secondary antibodies were: Alexa Fluor 488 goat anti-mouse IgG H+L (Thermo Fisher Scientific, A11001, 1:1000); Alexa Fluor 546 goat anti-mouse IgG1 (Thermo Fisher Scientific, A21123, 1:1000). Immunohistochemistry images were captured on a Zeiss LSM 800 or 900 laser scanning confocal microscope. Whole-mount (brightfield) images were captured using a Leica MZ 16F dissection microscope with a Retiga 4000RV camera. ImageJ (National Institutes of Health) was used for image analysis and standard image processing.

### Fluorescence *in situ* hybridization

We adapted a previously published protocol for the whole-mount *in situ* hybridization experiments (de Soysa et al., 2019). Briefly, embryos were fixed in 4% paraformaldehyde/PBST overnight at 4°C, washed three times with 1× PBST and dehydrated through 25%, 50%, 75% and 100% methanol. Embryos were stored in 100% methanol at −20°C until the *in situ* protocol was performed. The RNAscope Multiplex Fluorescent reagent Kit V2 (323100) was used according to the manufacturer's protocol with the following adaptations: the air-drying step was omitted, for embryos younger than E9.5 the protease digestion step was omitted, probes were hybridized overnight at 40°C, 0.2× SSCT (saline-sodium citrate with 0.01% Tween) was used for all wash steps and embryos were counterstained overnight with DAPI. Embryos were mounted onto coverslips and imaged in 1× PBST. For analysis of the FHF and SHF the entirety of the heart fields was imaged using a Zeiss LSM 900 laser scanning confocal microscope (Fig. 5). For *in situ* hybridization experiments performed on cryo-tissue sections, tissue was processed as described above. *In situ* hybridization was performed using the RNAscope Multiplex Fluorescent reagent Kit V2 (323100) according to the manufacturer's protocol, with the following adaptations: slides were thawed at room temperature for 5 min, the protease digestion step was omitted and probes were hybridized overnight at 40°C. The following RNAScope probes (ACDBio) were used in this study, *Hcn4* (421271), *Isl1* (451931-C3), *Tbx1* (481911), *Tbx5* (519581-C2) and *Aldh1a2* (540221-C3).

### Quantitation of cardiomyocyte proliferation

For quantification of cardiomyocyte proliferation, embryos were processed as described above (see ‘Histology and immunohistochemistry’). Cryosections were stained for TPM1, pHH3 and DAPI. For quantitative analysis, 11-16 sections were analyzed from control (Tbx20^+/+^) (*N*=3) and Tbx20^EH1mut^ (Tbx20^EH1Mut/EH1Mut^) (*N*=4) embryos. These sections represent the entirety of the heart (anterior, middle and posterior). The mitotic index was calculated by dividing the total cells positive for TMY and pHH3 by the total cells positive for TPM1. Statistical analysis was performed using Welch's *t*-test.

### Quantitation of the FHF and SHF population in CC

Analysis of the E7.75 embryos was performed using Imaris (10.0.01) software. Images were converted to Imaris file format and were masked to include only the CC. Surface volume renderings were performed using the Surface tool. The Spots tool was used to calculate the number of cells per surface and the number of *Hcn4*/*Isl1* double-positive cells. Statistical analysis was performed using Welch's *t*-test.

### Quantitation of Isl1^+^ cardiomyocytes and proliferating Isl1 cells in the SHF

For quantification of Isl1^+^ cardiomyocytes, embryos were processed as described above (see ‘Histology and immunohistochemistry’). Cryosections were co-stained for Isl1, SMA and DAPI. A total of seven sections corresponding to the anterior, middle and posterior positions of the heart were analyzed from wild-type (*N*=3) and Tbx20^EH1mut^ (*N*=3) embryos. Percentage Isl1^+^ cardiomyocytes were calculated by dividing total cells positive for SMA and Isl1 by total cells positive for SMA. To quantify the mitotic index of Isl1^+^ cells in the SHF, cryosections were co-stained with Isl1, pHH3 and DAPI. A total of 5-7 sections corresponding to anterior, middle and posterior positions of the SHF were analyzed from wild-type (*N*=4) and Tbx20^EH1mut^ (*N*=4) embryos. The mitotic index was calculated by dividing the total SHF cells positive for Isl1 and pHH3 by the total SHF cells positive for Isl1. Statistical analysis was performed using Welch's *t*-test.

### RNA-seq and analysis

E9.5 hearts were collected from 12 wild-type and 12 Tbx20^EH1Mut^ embryos. Four biological replicates were performed (three hearts were pooled for each replicate). RNA was isolated using the RNAqueous micro kit (Ambion) as per the manufacturer's protocol. Poly-A selected RNA-seq libraries preparation, sequencing reactions, and initial bioinformatic analysis were conducted at GENEWIZ, LLC. Samples were run on a HiSeq2500 (Illumina) with 2×150 bp paired-end reads.

Genes with an adjusted *P*-value<0.05 and a log2(fold change)>0.5 in either direction were considered differentially expressed. Canonical pathway and upstream regulator analysis were performed using Ingenuity Pathway Analysis (IPA) (Qiagen, https://digitalinsights.qiagen.com/products-overview/discovery-insights-portfolio/analysis-and-visualization/qiagen-ipa/). Tbx20 ChIP-seq data from E11.5 hearts was obtained from previously published data available from the European Molecular Biology Laboratory–European Bioinformatics Institute (EMBL-EBI) database (accession number E-MTAB-3967).

### Isolation of TBX20-GFP complexes for co-immunoprecipitation

TBX20 protein co-immunoprecipitation was performed using GFP-Trap magnetic beads (Chromotek) following the manufacturer's protocols with slight modification. Briefly, HEK293 cells expressing either wild-type (Tbx20-GFP) or Tbx20^EH1Mut^ (Tbx20^EH1MUT-GFP^) protein and Nkx2-5-V5 were washed twice with cold PBS (Thermo Fisher Scientific, 14190144), harvested by scraping with a cell lifter and centrifuged at 350 ***g*** for 10 min at 4°C. Cell pellets were resuspended in 600 µl lysis buffer [20 mM K-HEPES (pH 7.4), 150 mM NaCl, 100 mM KOAc, 2 mM MgCl_2_, 0.5% NP-40, 1 µm ZnCl_2_ 1 µm CaCl_2_] with protease (Millipore Sigma, 8340), phosphatase inhibitors (Millipore Sigma, 5726 and P0044) and nuclease (Thermo Fisher Scientific, 88700). The cell suspension was then incubated on ice for 30 min with pipetting every 10 min and then pelleted at 20,000 ***g*** for 10 min at 4°C. Then, 900 µl of dilution buffer [10 mM Tris-Cl (pH 7.5); 150 mM NaCl; 0.5 mM EDTA] was added to cleared lysate. Diluted lysate was incubated with 50 µl equilibrated GFP-Trap magnetic beads by rotating for 1 h at 4°C. Beads were washed three times with 500 μl of dilution buffer. GFP complexes were eluted from beads in 45 µl 6× Laemmli Sample Buffer at 95°C for 10 min. Protein complexes were examined by Western blotting.

## Supplementary Material

10.1242/develop.201677_sup1 Supplementary information

Table S1. Dataset of differentially expressed genes in wild-type and Tbx20^EH1Mut^ hearts at E9.5 from RNA-Seq analysis.
