# Supplementary material for: The Tbx20-TLE interaction is essential for the maintenance of the second heart field
Source: Development. 2023 Oct 30;150(21):dev201677. doi: 10.1242/dev.201677 (PMC10629681; doi:10.1242/dev.201677)
Supplement: Supplementary information [file develop-150-201677-s1.pdf]

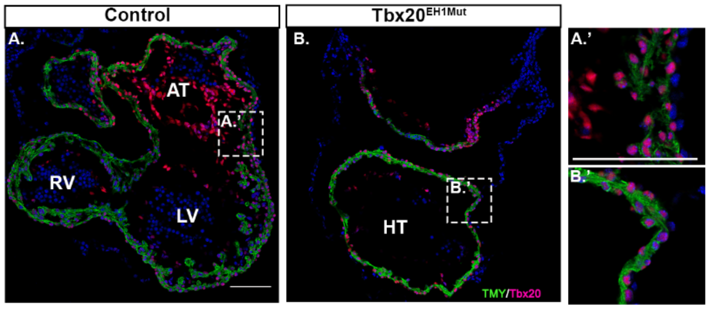

C.

Genotype	Stage	
	E9.5	PND0
$Tbx20^{+/+}$	31	5
	26.27%	31%
$Tbx20^{+/EH1Mut}$	57	11
	48.31%	69%
$Tbx20^{EH1Mut/EH1Mut}$	30	0
	25.42%	0%
N=	118	16

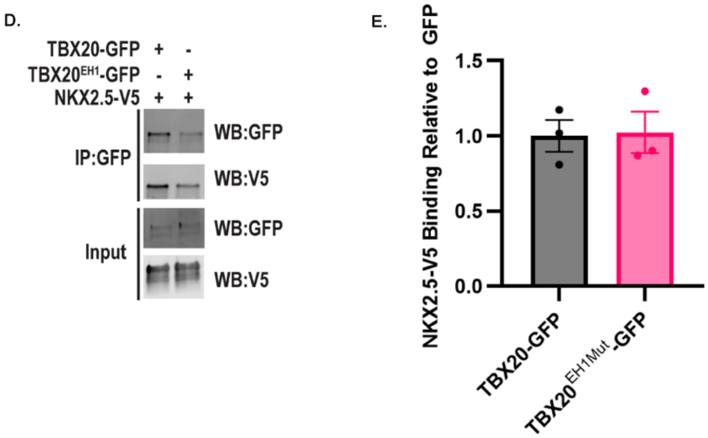

Fig. S1. Immunohistochemical analysis of Tbx20 in control and $Tbx20^{EH1Mut}$ embryos and genotype distribution. Related to Figure 1.

(A-B') Immunohistochemical analysis of Tbx20 (red) positive cardiomyocytes (TMY; green) in control and $Tbx20^{EH1Mut}$ hearts at E9.5. Scale bar, 100 μM.

(C) Genotype distribution of wild-type, heterozygous, and homozygous mutant embryos at E9.5 and postnatal day (PND) 0.

(D) Representative western blots demonstrating the interaction of Wild-type or $Tbx20^{EH1Mut}$ protein with Nkx2.5.

(E) Quantification of NKX2.5-V5 abundance in affinity-purified TBX20-GFP protein complexes. Protein abundance is relative to GFP. N = 3 biological replicates. Data expressed as means. Error bars represent ± SEM.

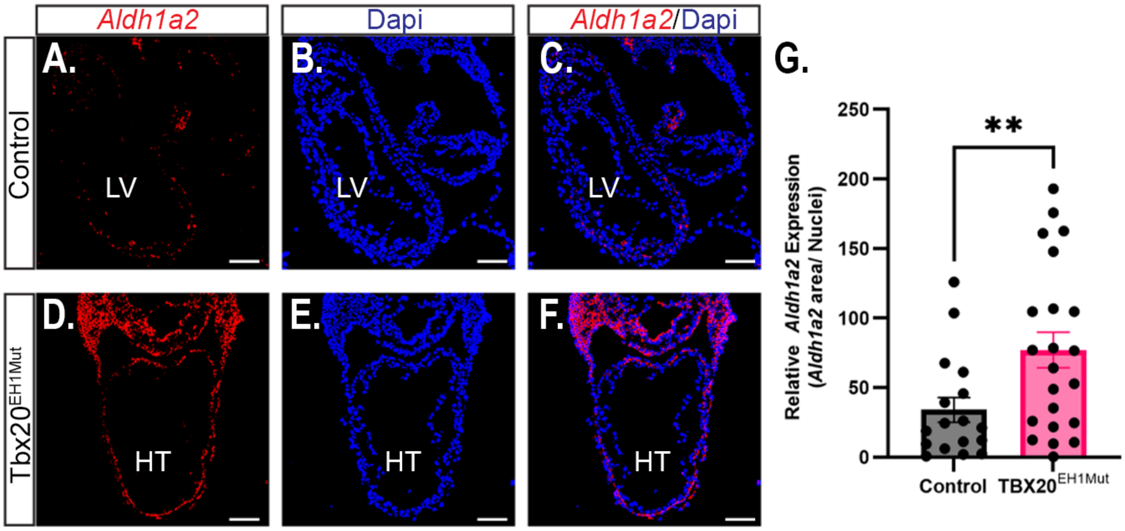

Fig. S2. RNA-FISH analysis of *Aldh1a2* in control and Tbx20^{EH1Mut} embryos at E9.5. Related to Figure 2.

(A-F) RNA-FISH analysis of *Aldh1a2* in the heart tube shows a significant increase in the expression of *Aldh1a2* in the heart tube of Tbx20^{EH1Mut} embryos at E9.5. Scale bar, 100uM.

(G) Quantitation of the total area of *Aldh1a2* expression in the heart tube of control and Tbx20^{EH1Mut} embryos. (N=3 per genotype, n=4-7 sections per animal). Data are expressed as mean values, error bars represent ± standard error of the mean (SEM). Welch's *t*-test, **p≤ 0.01

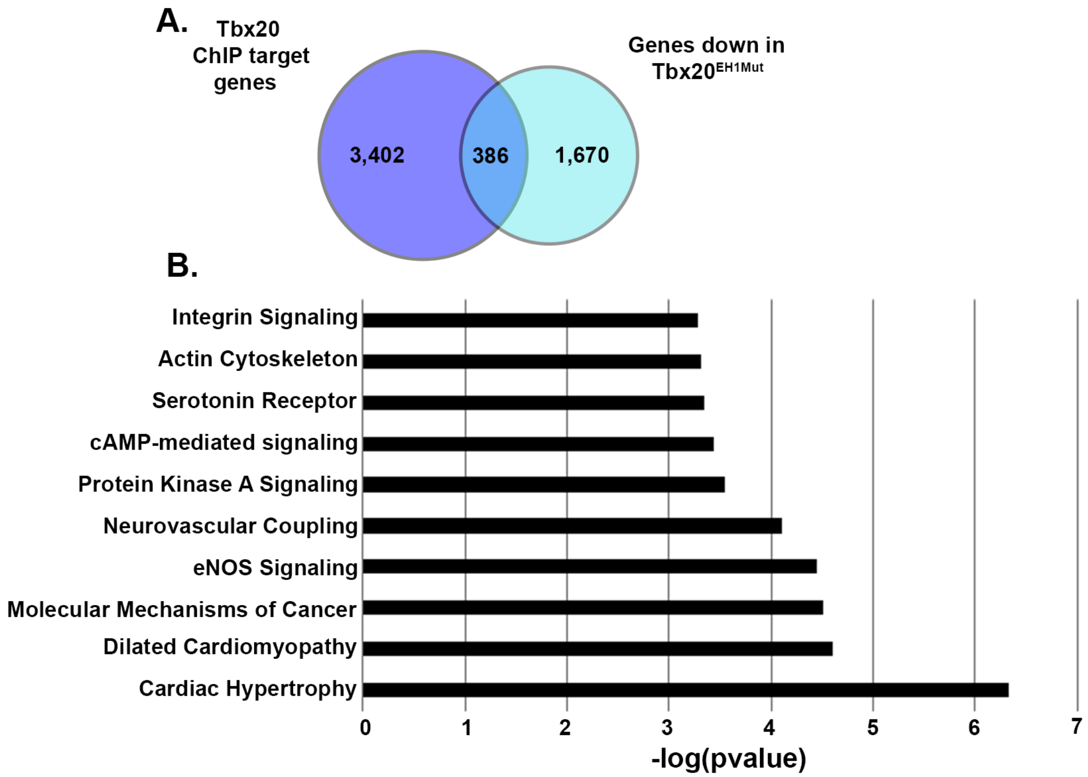

Fig. S3. Overlap of downregulated genes in Tbx20^{EH1Mut} hearts with Tbx20 ChIP-seq. Related to Figure 2.

(A) Overlap of downregulated genes in Tbx20^{EH1Mut} hearts (adjusted p-value ≤ 0.05 and log2 fold change ≥ 0.585) with Tbx20 ChIP-seq (chromatin immunoprecipitation followed by high throughput sequencing) dataset (Boogerd et al., 2016).

(B) Ingenuity Pathway Analysis of genes downregulated in Tbx20^{EH1Mut} hearts and identified as putative direct Tbx20 targets by Chip-Seq.

Development • Supplementary information

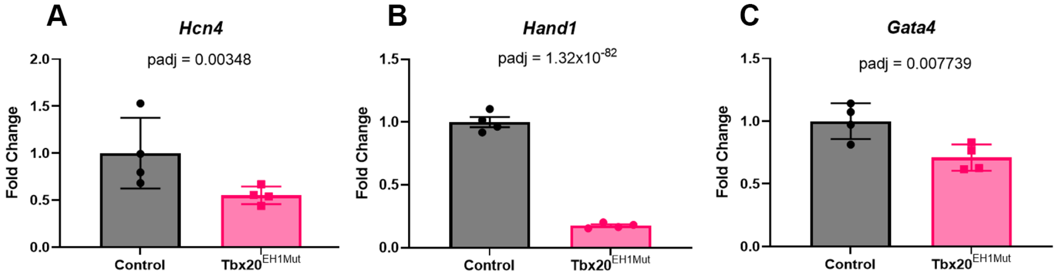

Fig. S4. Gene expression of First Heart Field associated genes in control and Tbx20^{EH1Mut} hearts, identified by RNA-Seq. Related to Figure 2.

(A-C) Relative gene expression analysis of first heart field associated genes in control and Tbx20^{EH1Mut} hearts. Data are shown as fold change. All samples normalized to average control expression.

Table S1. Dataset of differentially expressed genes in wild-type and Tbx20^{EH1Mut} hearts at E9.5 from RNA-Seq analysis.

Available for download at
https://journals.biologists.com/dev/article-lookup/doi/10.1242/dev.201677#supplementary-data
