# Supplementary material for: The Tbx20-TLE interaction is essential for the maintenance of the second heart field
Source: Development. 2023 Oct 30;150(21):dev201677. doi: 10.1242/dev.201677 (PMC10629681; doi:10.1242/dev.201677)
Supplement: Supplementary file 1 [file develop-150-201677_review_history.pdf]

The Tbx20-TLE interaction is essential for maintenance of the second heart field

Whitney Edwards, Olivia K. Bussey and Frank L. Conlon
DOI: 10.1242/dev.201677

Editor: Benoit Bruneau

Review timeline

Original submission:	3 February 2023
Editorial decision:	10 March 2023
First revision received:	31 August 2023
Accepted:	14 September 2023

Original submission

First decision letter

MS ID#: DEVELOP/2023/201677

MS TITLE: The Tbx20-TLE Interaction is Essential for Maintenance of the Second Heart Field

AUTHORS: Whitney Edwards, Olivia K. Bussey, and Frank L Conlon

I have now received all the referees' reports on the above manuscript, and have reached a decision. The referees' comments are appended below, or you can access them online: please go to BenchPress and click on the 'Manuscripts with Decisions' queue in the Author Area.

As you will see, the referees express considerable interest in your work, but have some significant criticisms and recommend a substantial revision of your manuscript before we can consider publication. All three Reviewers are agreed on: 1) showing some data on earlier FHF/SHF populations (and perhaps RA signaling targets in posterior vs anterior SHF); 2) discussing the FHF vs SHF phenotypes to a greater extent; 3) comparing the Tbx20 null and point mutant phenotypes (at least in the Discussion), and two suggested intersecting ChIPseq data. Other points should also be addressed. If you are able to revise the manuscript along the lines suggested, which may involve further experiments, I will be happy receive a revised version of the manuscript. Your revised paper will be re-reviewed by one or more of the original referees, and acceptance of your manuscript will depend on your addressing satisfactorily the reviewers' major concerns. Please also note that Development will normally permit only one round of major revision. If it would be helpful, you are welcome to contact us to discuss your revision in greater detail. Please send us a point-by-point response indicating your plans for addressing the referee’s comments, and we will look over this and provide further guidance.

Please attend to all of the reviewers' comments and ensure that you clearly highlight all changes made in the revised manuscript. Please avoid using 'Tracked changes' in Word files as these are lost in PDF conversion. I should be grateful if you would also provide a point-by-point response detailing how you have dealt with the points raised by the reviewers in the 'Response to Reviewers' box. If you do not agree with any of their criticisms or suggestions please explain clearly why this is so.

© 2023. Published by The Company of Biologists under the terms of the Creative Commons Attribution License (https://creativecommons.org/licenses/by/4.0/).

Reviewer 1

Advance summary and potential significance to field

In this manuscript, Edwards and colleagues use a mouse model to examine the function of the Tbx20-TLE (Transducin-Like Enhancer of Split, Groucho in Drosophila) interaction in cardiac development. Tbx20 is a well-characterized cardiac transcription factor with multiple roles in heart development, and is further a known player in congenital heart abnormalities. Tbx20 function is likely carried out by interactions with multiple transcription factors and transcriptional co-activators and -repressors, however for the most part the in vivo role of these characterized in vitro interactions have not been assessed.

A sophisticated and precise targeted Tbx20 mutant, in which 2 amino acids are changed in a motif previously shown by this group to be essential for the Tbx20-TLE interaction (and which had functional consequences in Xenopus development), was engineered via CRISPR/Cas9 genome editing. Homozygous (but not heterozygous) “Tbx20 EH1” mutants presented with severe cardiac development defects by e9.5 with an absence of cardiac looping and formation of many chamber elements and a significant reduction in cardiomyocyte number. Gene expression analysis (bulk RNAseq) of e9.5 hearts was used to demonstrate down-regulation of many cardiomyocyte and proliferation associated genes in EH1 mutants, and upregulation of genes associated with retinoic acid (RA) signalling, with these putative RA targets matching well with published Tbx20 ChIPseq data. Upregulation of described cardiac progenitor/SHF and outflow tract associated genes is also apparent in EH1 mutants, with Isl1 expression now apparent throughout the heart tube. In EH1 mutants a decreased Isl1+ SHF population is evident at e9.5, with decreased proliferation in this population. While SHF contribution to the heart is still apparent (Mef2c:Cre lineage trace), it is reduced in magnitude.

Overall, these results highlight the importance of the Tbx20-TLE interaction in regulating gene expression programs critical for proper maintenance of the SHF progenitor population and likely differentiation of cardiomyocytes within the e9.5 heart tube. This functions may be in part mediated via regulation of RA signalling, a known regulator of SHF development.

Overall, this is a concise and well-written manuscript with in general well presented experimental data. The importance of the Tbx20-TLE interaction is clearly presented.

Comments for the author

Minor points:

1. It should be discussed why Tbx20 EH1 hets do not have an apparent phenotype. One may expect that this form of Tbx20 could have a dominant, “gain-of-function” activity.

2. The severity of the Tbx20 EH1 and Tbx20 null phenotypes should be compared directly, at least in the Discussion. As the EH1 mutants have a severe phenotype, is a major role of Tbx20 to act as a repressor via TLE recruitment?

Major points:

1. Effects on the balance of FHF vs SHF specification are alluded to in the discussion. These could be directly assessed by histological and RNA ISH/IF analysis at earlier stages of cardiogenesis.

2. It is somewhat confusing how SHF-related genes appear to be upregulated in the heart tube of mutants yet the SHF population itself appears to be reduced (at least based on Isl1 staining). As SHF contribution to the heart (which may be expected to deplete the progenitor pool) does not appear to be enhanced, the authors should address this further, at least in the Discussion, or perhaps via direct analysis of other SHF markers.

© 2023. Published by The Company of Biologists under the terms of the Creative Commons Attribution License (https://creativecommons.org/licenses/by/4.0/).

Reviewer 2

Advance summary and potential significance to field

Edwards et al have investigated the functional significance of the interaction between Tbx20 and TLE repressors in vivo, revealing roles during early heart development. Using gene editing the authors altered two critical amino acids of Tbx20, leading to shifts in cardiac progenitor cell expression consistent with an increase in second and decrease in first heart field markers and altered progenitor cell patterning, including increased expression of genes in the retinoic acid pathway. This clearly written manuscript provides new insights into the molecular mechanisms by which an important T-box regulator functions in early heart development. However, addressing the following points would help to reinforce the authors' conclusions.

Comments for the author

1. The authors show that the gene edited mutation has a striking early cardiac phenotype. It would be helpful to provide further information as to how closely this phenotype compares with reported Tbx20 null phenotypes. Do the new results rule out roles independent of the repressor complex interaction?

2. Given the similarity with Nkx2-5 mutant hearts where progenitor genes such as Isl1 are upregulated, can the authors confirm that the mutant version of Tbx20 is still able to interact with Nkx2-5?

3. The authors show conservation of nuclear Tbx20 accumulation in mutant cardiomyocytes (supplemental Figure 1). However there appears to be either an endodermal defect or loss of Tbx20 expression in endoderm that might have a major impact on the phenotype. The authors should comment on this point.

4. The downregulation of first heart field genes is particularly interesting. Can the authors provide any spatial expression data for Hcn4, Gata4 or Hand1? Analysis of Tbx5 expression in mutant hearts would clearly be helpful in addressing this point.

5. Please indicate whether the downregulated genes also include Tbx20 ChIP predicted targets.

6. The authors conclusions would be significantly reinforced if they could provide in situ or immunofluorescence data showing increase in the expression levels or domains of genes in the RA pathway emerging from their RNA sequencing data (such as Aldh1a2, Hox1, Crabp1).

7. In Figure 4F the aSHF label appears to indicate Isl1 expression in ventral foregut endoderm.

Minor points:

8. Please clarify the term "systematically" as used in the abstract.

9. In Figure 3 the color shadings for Isl1 and Nnat differ between panels B and D, yet should presumably be identical.

10. Line 165: Rbp1

Reviewer 3

Advance summary and potential significance to field

Edwards et al. investigated the role of Tbx20 in the developing heart in this manuscript, focusing specifically on the interaction of Tbx20 with transducing-like enhancer of split (TLE). The Tbx20-TLE complex represses transcription of many progenitor genes, allowing the cardiac progenitor cells to differentiate properly and the heart to develop and loop properly. By introducing a two amino

© 2023. Published by The Company of Biologists under the terms of the Creative Commons Attribution License (https://creativecommons.org/licenses/by/4.0/).

acid mutation to the EH1 domain, where Tbx20 and TLE interact, cardiac looping and chamber development is disrupted, resulting in embryonic lethality.

Transcriptomics analysis revealed that loss of the Tbx20-TLE interaction led to dysregulation of retinoic acid genes and cardiomyocyte cell cycle and growth genes. Further analysis showed that the second heart field progenitors are lost which contributes to the impaired development seen in these animals. Overall the manuscript is well-written and presents a convincing phenotype and explanation of the underlying mechanisms. However, we believe that there are several points to address and further clarify prior to publication.

Comments for the author

1. Disrupting the Tbx20-TLE interaction results in both upregulated and downregulated genes. The authors claim that Tbx20-TLE forms a repressive complex. Therefore, they should comment on the regulation of these upregulated AND downregulated genes, specifically direct vs indirect regulation. For example, in the cardiac cell cycle and growth gene sets, are there Tbx20 binding sites at the promoter or enhancer regions, or is there indirect regulation of these genes via de-repression of a repressor of these genes? The authors have both Tbx20 RNA-seq and ChIP-seq data to address this.

2. Along similar lines, is Tbx20 binding or chromatin accessibility/conformation or DNA/histone methylation affected by EH1 domain mutation? Are the transcriptional differences in the mutant vs control due to the loss of transcriptional cofactors in the Tbx20-TLE complex? Is it known if the recruitment of the NuRD components is affected?

3. The finding that loss of the EH1 domain interaction leads to an imbalance of FHF and SHF populations is novel, interesting, and should be explored further, at least in the discussion. How might the Tbx20-TLE complex affect FHF and SHF differentially (Tbx20 is also expressed in the FHF, yet the mutants have an expanded SHF population)?

4. Since the WT hearts have higher Tbx20 in the pSHF compared to the aSHF is it possible to examine how each SHF subpopulations are affected by the EH1 mutation? Using markers of each population

5. The authors should further elaborate at least in the writing on the findings that there are more Isl1+ CMs in the heart tube (Figure 3) but less in the SHF (Figure 5) at E9.5 in mutant vs control. How does the SHF progenitors migrate to the heart tube in the mutant? What gene programs might be dysregulated to result in this phenotype? Is this related with the spatio-temporal pattern of Tbx20 expression?

6. The authors should comment on the finding that the EH1 mutant results in the cells staying in a progenitor state, yet losing the ability to go through cell cycle. Are any subpopulations only affected by one (differentiation blockage vs cell cycle blockage) or are they all affected by both? In a normal WT heart, what is the interplay between differentiation and cell cycle progression?

7. The addition of a model summarizing the findings of the manuscript would be helpful for readers.

First revision

Author response to reviewers' comments

Reviewer 1 Comments for the Author:

Major points:

1. Effects on the balance of FHF vs. SHF specification are alluded to in the discussion. These could

© 2023. Published by The Company of Biologists under the terms of the Creative Commons Attribution License (https://creativecommons.org/licenses/by/4.0/).

be directly assessed by histological and RNA ISH/IF analysis at earlier stages of cardiogenesis.
Response: To address Reviewer #1's comment, we have performed additional analyses of the FHF and SHF in control and Tbx20^{EH1Mut} embryos. These studies include:

1. Studies of the FHF (*Hcn4*+) and SHF (*Isl1*+) cardiac progenitor populations in control and Tbx20^{EH1Mut} embryos at E7.75 using whole mount in situ hybridization (new Figures 5A-5J).
2. Through the generation of 3D projections of the developing heart fields using confocal imaging, detailed spatial and quantitative analyses of FHF and SHF progenitors in control and Tbx20^{EH1Mut} hearts (new Figure 5C, 5D, 5G and 5H).

Consistent with our initial findings, these studies show altered localization and organization of SHF progenitors in Tbx20^{EH1Mut} embryos compared to controls (new Figures 5C and 5G). In addition, we demonstrate in our revised submission that the number of *Hcn4* and *Isl1* double- positive cells are dramatically increased in Tbx20^{EH1Mut} embryos, suggesting that Tbx20^{EH1Mut} hearts is due to the misspecification of cardiac progenitors (new Figures 5D, 5H, 5K, and 5L).

2. It is somewhat confusing how SHF-related genes appear to be upregulated in the heart tube of mutants, yet the SHF population itself appears to be reduced (at least based on Isl1 staining). As SHF contribution to the heart (which may be expected to deplete the progenitor pool) does not appear to be enhanced, the authors should address this further, at least in the Discussion, or perhaps via direct analysis of other SHF markers.

Response: We agree with the reviewer and regret our presentation was not more lucid. We have addressed the issue in the revised submission. In summary, we propose that the reduction of the SHF pool paralleled the increase in the proportion of SHF-derived cells in the heart tube at E9.5. Thus implying premature deployment of SHF progenitors into the developing heart tube. Though these SHF-derived cells are incorporated into the heart tube, the progenitor cardiac cells are arrested in development and, therefore, cannot differentiate. To further clarify our hypothesis, we expanded our Discussion and have now included a schematic of our proposed model (new Figure 7). Also, please see the response to Reviewer #3, Point 2.

Minor points:

1. It should be discussed why Tbx20 EH1 hets do not have an apparent phenotype. One may expect that this form of Tbx20 could have a dominant, "gain-of-function" activity.

Response: In fly, c. elegans, zebrafish, and Xenopus, it has been demonstrated that mutations in the EH1 domain result in loss of function with no reported phenotype in heterozygous animals (PMID: 14561704, PMID: 16314497, PMID: 24024827, PMID: 21852953, PMID: 21852953, PMID: 24024827). Consistently, we have formally demonstrated in previous work (PMID: 24024827) that the EH1 mutation in TBX20 (Tbx20^{EH1Mut}) acts as a loss of function mutation in cell lines and Xenopus. In response to Reviewer #1's comment, we have emphasized these findings in our revised text.

2. The severity of the Tbx20 EH1 and Tbx20 null phenotypes should be compared directly, at least in the Discussion. As the EH1 mutants have a severe phenotype, is a major role of Tbx20 to act as a repressor via TLE recruitment?

Response: The reviewer bring up a salient point. In response to the reviewer's comments and that of Reviewer #2, we now include a direct comparison of the reported Tbx20 mouse null phenotypes to our Tbx20^{EH1Mut} allele in the revised Discussion.

Reviewer 2 Comments for the Author:

1. The authors show that the gene-edited mutation has a striking early cardiac phenotype. It would be helpful to provide further information as to how closely this phenotype compares with reported Tbx20 null phenotypes. Do the new results rule out roles independent of the repressor

© 2023. Published by The Company of Biologists under the terms of the Creative Commons Attribution License (https://creativecommons.org/licenses/by/4.0/).

complex interaction?

Response: In response to Reviewers #1 and #2, we have expanded our Discussion to directly compare the published mouse Tbx20 null phenotypes with our Tbx20^{EH1Mut} allele. Also, please see the response to Reviewer #1, Minor point 2.

2. Given the similarity with Nkx2-5 mutant hearts where progenitor genes such as Isl1 are upregulated, can the authors confirm that the mutant version of Tbx20 can still interact with Nkx2-5?

Response: To address the reviewer's comment, we assessed if mutating the EH1 domain affects the interaction between Tbx20 and Nkx2-5. Co-immunoprecipitation experiments were performed with either wildtype (Tbx20-GFP) or Tbx20^{EH1Mut} (Tbx20^{EH1Mut}-GFP) protein and Nkx2- 5-V5 in HEK293 cells. We did not observe differences in co-isolation of Nkx2-5 with wildtype or mutant protein. These findings suggest that disruption of the Tbx20/TLE repression complex does not affect the interaction between Tbx20 and Nxk2-5. These results are now included in the Results and as new Supplemental Figure #1.

3. The authors show the conservation of nuclear Tbx20 accumulation in mutant cardiomyocytes (Supplemental Figure 1). However, there appears to be either an endodermal defect or loss of Tbx20 expression in the endoderm that might have a significant impact on the phenotype. The authors should comment on this point.

Response: We thank the reviewer for the comment and have clarified this point.

4. The downregulation of first heart field genes is particularly interesting. Can the authors provide spatial expression data for Hcn4, Gata4 or Hand1?Analysis of Tbx5 expression in mutant hearts would clearly be helpful in addressing this point.

Response: To address the Reviewer's #2 comments, we substantially expanded our analysis of the FHF and SHF progenitors in control and Tbx20^{EH1Mut} embryos. For our new studies, we assessed the FHF (*Hcn4+*) and SHF (*Isl1+*) cardiac progenitor populations in control and Tbx20^{EH1Mut} embryos at E7.75 using whole mount in situ hybridization (new Figures 5A-5J). In addition, we analyzed the expression of Tbx5 in the pSHF by fluorescence in situ hybridization at E9.5 (new Figures 6I and 6J). Also, please see the response to Reviewer #1, Point 1.

5. Please indicate whether the downregulated genes also include Tbx20 ChIP predicted targets.

Response: We regret this data was not included in the initial submission. In our revised submission, we have intersected the downregulated genes identified in our RNA-seq analysis with previously published Tbx20-ChIP-seq data. Our new studies showed there are 386 downregulated genes with putative Tbx20 binding sites (Discussed in Figure 2 results section and new Supplemental Figure 3A). This subset of genes is associated with myocardial chamber formation, growth, and cardiomyocyte energy metabolism and contractility (new Supplemental 3B). However, it is unclear if these changes in gene expression are a direct result of alterations in the Tbx20-TLE complex or a result of impaired chamber development and maturation of the heart tube observed in Tbx20^{EH1Mut} embryos.

6. The authors conclusions would be significantly reinforced if they could provide in situ or immunofluorescence data showing an increase in the expression levels or domains of genes in the RA pathway emerging from their RNA sequencing data (such as Aldh1a2, Hox1, Crabp1).

Response: We appreciate the reviewer’s suggestion and have now validated the increased expression of *Aldh1a2* in Tbx20^{EH1Mut} hearts at E9.5 by fluorescence in situ hybridization (new Supplemental Figure 2A-2G).

7. In Figure 4F, the aSHF label appears to indicate Isl1 expression in ventral foregut endoderm.

© 2023. Published by The Company of Biologists under the terms of the Creative Commons Attribution License (https://creativecommons.org/licenses/by/4.0/).

Response: We thank the reviewer and have corrected the labeling in Figure 4F.

Minor points:

8. Please clarify the term "systematically" as used in the abstract.

Response: The term systematically has been removed from the abstract.

9. In Figure 3 the color shadings for Isl1 and Nnat differ between panels B and D yet should presumably be identical.

Response: We regret our error and have corrected the labeling for the heatmaps in Figure 3 Panel D.

10. Line 165: Rbp1

Response: We have corrected this gene name.

Reviewer 3 Comments for the Author:

1. Disrupting the Tbx20-TLE interaction results in both upregulated and downregulated genes. The authors claim that Tbx20-TLE forms a repressive complex. Therefore, they should comment on the regulation of these upregulated AND downregulated genes, specifically direct vs. indirect regulation. For example, in the cardiac cell cycle and growth gene sets, are there Tbx20 binding sites at the promoter or enhancer regions, or is there indirect regulation of these genes via de-repression of a repressor of these genes? The authors have both Tbx20 RNA-seq and ChIP-seq data to address this.

Response: In response to Reviewers #2 and #3, we intersected the downregulated genes identified from our RNA-seq analysis with previously published Tbx20-ChIP-seq data. Please see the response to Reviewer #2, Point 5.

2. Along similar lines, is Tbx20 binding or chromatin accessibility/conformation or DNA/histone methylation affected by EH1 domain mutation? Are the transcriptional differences in the mutant vs. control due to the loss of transcriptional cofactors in the Tbx20-TLE complex? Is it known if the recruitment of the NuRD components is affected?

Response: We regret not elaborating on our previous studies in which we have characterized the TBX20 and the $Tbx20^{EH1Mut}$ interactomes (PMID: 24024827). These published findings conclude:

1. $Tbx20^{EH1Mut}$ disrupted the interaction of TBX20 with components of the TLE and NuRD complexes (e.g., Rbbp4, Rbbp7, Mta1, and Hdac2).
2. That $Tbx20^{EH1Mut}$ acts as a loss of function mutation; no new proteins were shown to interact/complex with $Tbx20^{EH1Mut}$ versus TBX20.
3. There are no changes in the relative affinity for the remaining components of the $Tbx20^{EH1Mut}$ interactome relative to the TBX20 interactome.
4. $Tbx20^{EH1Mut}$ loses the ability to repress direct targets of wildtype TBX20 in Xenopus bioassays. Moreover, no new transcriptional activation activity was identified.

Based on our new data and these findings, we hypothesize that chromatin accessibility and complex cofactor recruitment are altered in the $Tbx20^{EH1Mut}$ embryos. To test this hypothesis would require ChIP-Seq analysis and immunoprecipitation mass spectrometry (IPMS) from $Tbx20^{EH1Mut}$ hearts. Given that the embryos die shortly after E10.5, these types of studies are not technically feasible due to 1) the small amounts of tissue, 2) the absence of validated ChiP/ IPMS-compatible Tbx20 antibodies, and 3) it would be difficult to discern given the $Tbx20^{EH1Mut}$ phenotype if any alterations we observe are direct or indirect.

© 2023. Published by The Company of Biologists under the terms of the Creative Commons Attribution License (https://creativecommons.org/licenses/by/4.0/).

3. The finding that loss of the EH1 domain interaction leads to an imbalance of FHF and SHF populations is novel interesting, and should be explored further, at least in the discussion. How might the Tbx20-TLE complex affect FHF and SHF differentially (Tbx20 is also expressed in the FHF, yet the mutants have an expanded SHF population)?

Response: Regarding Reviewer #3's comment, we have performed additional studies on the FHF and SHF in control and Tbx20^{EH1Mut} embryos at E7.75. In response, we have:

1. Characterized the FHF (*Hcn4*+) and SHF(*Isl1*+) cardiac progenitor populations in control and Tbx20^{EH1Mut} embryos at E7.75 using whole mount in situ hybridization (new Figures 5A-5J).
2. Generated 3D projections of the developing heart fields and performed spatial and quantitative analyses of FHF and SHF progenitors (new Figure 5C, 5D, 5G, and 5H).
3. Showed an altered localization and organization of SHF progenitors in Tbx20^{EH1Mut} embryos compared to controls (new Figures 5C and 5G).
4. Showed a dramatic increase in the number of Hcn4 and Isl1 double-positive cells in Tbx20^{EH1Mut} embryos, thus further supporting our conclusions suggesting misspecification of cardiac progenitors (new Figures 5D, 5H, 5K, and 5L).

4. Since the WT hearts have higher Tbx20 in the pSHF compared to the aSHF, is it possible to examine how each SHF subpopulations is affected by the EH1 mutation? Using markers of each population

Response: As suggested, we analyzed the posterior and anterior SHF in control and Tbx20^{EH1Mut} embryos at E9.5 by the expression of Tbx1 (aSHF) and Tbx5 (pSHF) using fluorescence in situ hybridization. Our results show that while the boundaries between the aSHF and pSHF are maintained, both genes' overall expression decreases in the mutants (new Figures 6I and 6J). Consistently, the relative length of the Tbx1 and Tbx5 domains are significantly reduced in Tbx20^{EH1Mut} embryos compared to controls (new Figures 6K and 6L). Thus, these studies support our initial conclusions, suggesting that loss of the Tbx20-TLE complex results in perturbation of both aSHF and pSHF progenitor populations.

5. The authors should further elaborate, at least in the writing, on the findings that there are more Isl1+ CMs in the heart tube (Figure 3) but less in the SHF (Figure 5) at E9.5 in mutant vs. control. How does the SHF progenitors migrate to the heart tube in the mutant? What gene programs might be dysregulated to result in this phenotype? Is this related with the spatio-temporal pattern of Tbx20 expression?

Response: We thank the reviewer and have expanded our discussion to this point. In summary, our new analysis of the FHF and SHF cardiac progenitors at early stages of heart development provides new insights into the phenotype observed in the Tbx20^{EH1Mut} allele (new Figure 5). We hypothesize that impaired heart tube formation in mutants is due in part to compromised progenitor specification, which results in uncoordinated differentiation and deployment. We propose that the reduction of the SHF pool, paralleled with the increase in the proportion of SHF-derived cells in the heart tube at E9.5, suggests premature deployment of SHF progenitors into the developing heart tube. Further, although these SHF-derived cells are incorporated into the heart tube, they are arrested in development and cannot differentiate. To further clarify, we expanded our discussion to include this proposed explanation. We have incorporated a proposed model addressing the underlying mechanisms contributing to the cardiac phenotype observed in Tbx20^{EH1Mut} (new Figure 7).

6. The authors should comment on the finding that the EH1 mutant results in the cells staying in a progenitor state yet losing the ability to go through the cell cycle. Are any subpopulations only affected by one (differentiation blockage vs. cell cycle blockage), or are they all affected by both? In a normal WT heart, what is the interplay between differentiation and cell cycle progression?

Response: We agree with the reviewer that the apparent increase in cells in a progenitor-like state

© 2023. Published by The Company of Biologists under the terms of the Creative Commons Attribution License (https://creativecommons.org/licenses/by/4.0/).

paralleled with a decrease in cardiomyocyte proliferation is paradoxical. The dynamics of cardiomyocyte proliferation are rapidly changing during the early stages of embryonic heart development. Early cardiac progenitors display robust proliferation, but cardiomyocyte proliferation ceases as cells begin to differentiate during primary heart tube formation. Proliferation is then reinitiated after looping and in the beginning stages of chamber formation (Günthel et al., 2018; van den Berg et al., 2009 (PMC or PMIDs unavailable)). Because we observe impaired looping/chamber formation, we propose that the decrease in proliferation observed in $Tbx20^{EH1Mut}$ may reflect an inability to reinitiate cardiomyocyte proliferation. These points have been included in our revised discussion.

7. The addition of a model summarizing the findings of the manuscript would be helpful for readers.

Response: In response to the reviewer's comments, we have expanded our discussion and have included a model summarizing our findings (new Figure 7).

Second decision letter

MS ID#: DEVELOP/2023/201677

MS TITLE: The Tbx20-TLE Interaction is Essential for Maintenance of the Second Heart Field

AUTHORS: Whitney Edwards, Olivia K. Bussey, and Frank L Conlon
ARTICLE TYPE: Research Article

I am happy to tell you that your manuscript has been accepted for publication in Development, pending our standard ethics checks.

Reviewer 1

Advance summary and potential significance to field

In this manuscript, Edwards and colleagues use a mouse model to examine the function of the Tbx20-TLE (Transducin-Like Enhancer of Split, Groucho in Drosophila) interaction in cardiac development. Tbx20 is a well-characterized cardiac transcription factor with multiple roles in heart development, and is further a known player in congenital heart abnormalities. Tbx20 function is likely carried out by interactions with multiple transcription factors and transcriptional co-activators and -repressors, however for the most part the in vivo role of these characterized in vitro interactions have not been assessed.

A sophisticated and precise targeted Tbx20 mutant, in which 2 amino acids are changed in a motif previously shown by this group to be essential for the Tbx20-TLE interaction (and which had functional consequences in Xenopus development), was engineered via CRISPR/Cas9 genome editing. Homozygous (but not heterozygous) "Tbx20 EH1" mutants presented with severe cardiac development defects by e9.5 with an absence of cardiac looping and formation of many chamber elements and a significant reduction in cardiomyocyte number. Gene expression analysis (bulk RNAseq) of e9.5 hearts was used to demonstrate down-regulation of many cardiomyocyte and proliferation associated genes in EH1 mutants, and upregulation of genes associated with retinoic acid (RA) signalling, with these putative RA targets matching well with published Tbx20 ChIPseq data.
Upregulation of described cardiac progenitor/SHF and outflow tract associated genes is also apparent in EH1 mutants, with Isl1 expression now apparent throughout the heart tube. In EH1 mutants a decreased Isl1+ SHF population is evident at e9.5, with decreased proliferation in this population. While SHF contribution to the heart is still apparent (Mef2c:Cre lineage trace), it is reduced in magnitude.

© 2023. Published by The Company of Biologists under the terms of the Creative Commons Attribution License (https://creativecommons.org/licenses/by/4.0/).

Overall, these results highlight the importance of the Tbx20-TLE interaction in regulating gene expression programs critical for proper maintenance of the SHF progenitor population and likely differentiation of cardiomyocytes within the e9.5 heart tube. This functions may be in part mediated via regulation of RA signalling, a known regulator of SHF development. This is a concise and well-written manuscript with in general well presented experimental data. The importance of the Tbx20-TLE interaction is clearly presented.

Comments for the author

The authors have addressed my comments. The addition of the model schematic and further discussion of the status of prematurely differentiated SHF cells in the heart is appreciated.

Reviewer 2

Advance summary and potential significance to field

The authors have added significant experimental data to their interesting manuscript, fully addressing my previous concerns. This study provides important new insights into how Tbx20 regulates early heart development, highlighting a key role for transcriptional repression.

Minor point: A typo in the new model figure needs correction (disrupted).

Comments for the author

Minor point: A typo in the new model figure needs correction (disrupted).

Reviewer 3

Advance summary and potential significance to field

The authors went above and beyond in their revisions, and addressed all of the comments of the other reviewers and myself in a satisfactory manner. I have no additional comments.

Comments for the author

No additional comments.

© 2023. Published by The Company of Biologists under the terms of the Creative Commons Attribution License (https://creativecommons.org/licenses/by/4.0/).
